# Lockbox enrichment facilitates manipulative and cognitive activities for mice

**DOI:** 10.12688/openreseurope.17624.1

**Published:** 2024-06-04

**Authors:** Katharina Hohlbaum, Niek Andresen, Paul Mieske, Pia Kahnau, Benjamin Lang, Kai Diederich, Rupert Palme, Lars Mundhenk, Henning Sprekeler, Olaf Hellwich, Christa Thöne-Reineke, Lars Lewejohann

**Affiliations:** 1 German Centre for the Protection of Laboratory Animals (Bf3R), German Federal Institute for Risk Assessment (BfR), Berlin, 12277, Germany; 2 Science of Intelligence, Research Cluster of Excellence, Berlin, 10587, Germany; 3 Institute of Animal Welfare, Animal Behavior and Laboratory Animal Science, School of Veterinary Medicine, Freie Universitat Berlin, Berlin, 14163, Germany; 4 Computer Vision and Remote Sensing, Technische Universitat Berlin, Berlin, 10587, Germany; 5 Experimental Endocrinology, Department of Biological Sciences and Pathobiology, University of Veterinary Medicine, Vienna, 1210, Austria; 6 Institute of Veterinary Pathology, School of Veterinary Medicine, Freie Universitat Berlin, Berlin, 14163, Germany; 7 Modeling of Cognitive Processes, Technische Universitat Berlin, Berlin, 10587, Germany

**Keywords:** Mice, refinement, animal welfare, enrichment, home cage, cognition, sequential problem-solving, phenotyping

## Abstract

**Background:**

Due to the lack of complexity and variety of stimuli, conventional housing conditions of laboratory mice do not allow these animals to fully express their behavioral repertoire, including manipulative and cognitive activities. Therefore, we designed mechanical puzzles, so-called lockboxes, for mice that can be provided in their home cages. We investigated the impact of the lockbox enrichment on their phenotype and affective state when compared to conventional housing and super-environmental enrichment.

**Methods:**

Young adult female C57BL/6JCrl mice were examined before and after 2-month exposure to the different types of enrichment in a phenotyping test battery, including tests for trait and state anxiety-related behavior, calorimetric measurements, body weight measurements, and the analysis of stress hormone metabolite concentrations as well as sequential problem-solving abilities. At the end of the study, adrenal gland weights were determined and pathohistological evaluation was performed. For all continuous variables, the relative variability was calculated.

**Results:**

We demonstrated that lockbox enrichment decreased trait anxiety-related behavior compared to conventional housing in the Grid Exploratory Paradigm. In contrast, the different types of enrichment neither influenced state anxiety-related behavior in the Open Field and Elevated Plus Maze Test nor physiological variables (i.e., bodyweight, resting metabolic rate, stress hormone metabolite concentrations, and adrenal gland weights). Lockbox enrichment improved sequential problem-solving when compared to super-environmental enrichment. Regardless of the housing condition, the relative variability increased in most variables over time, although the coefficient of variation decreased for some variables, especially in animals with access to the lockbox enrichment. A pathohistological evaluation revealed no evidence of toxicopathological effects associated with the material from which the lockbox enrichment was made.

**Conclusions:**

Overall, the lockbox enrichment revealed beneficial effects on the affective state and sequential problem-solving abilities of laboratory mice. The reproducibility was demonstrated not to be compromised by lockbox enrichment, similar to super-environmental enrichment.

## Introduction

In the wild, mice live in complex environments that offer a variety of stimuli. In comparison to natural habitats, the laboratory environment is restricted in space, complexity, and stimuli, preventing the mice from fully expressing their behavioral repertoire ^1^. This can diminish the health and well-being of animals, thereby potentially affecting the quality of research data ^2^. Therefore, the European legislation stipulates that “all animals shall be provided with space of sufficient complexity to allow expression of a wide range of normal behaviour. […] Establishments shall have appropriate enrichment techniques in place, to extend the range of activities available to the animals and increase their coping activities including physical exercise, foraging, manipulative and cognitive activities, as appropriate to the species” ^3^.

Within the past decades, a range of different physical/inanimate environmental enrichment items have been introduced to enhance complexity in the laboratory environment ^1, 4– 6^. Additionally, it was proposed to house mice within a semi-natural environment ^6, 7^ and in larger social groups ^7^. In contrast, enrichment strategies for laboratory mice that specifically allow manipulative and cognitive activities have been rarely reported. The following examples can be found when searching for “cognitive enrichment” (search term in PubMed: "cognitive enrichment"[All Fields] AND ("mice"[MeSH Terms] OR "mice"[All Fields])): toys of different shapes, sizes, colors, and textures that do not increase physical activity (no further information given in the references) ^8^, multi-colored blocks, balls, and tunnels ^9^, positive reinforcement training (i.e., clicker training) ^10^, touchscreen training ^11– 13^, other operant learning cognitive tasks in operant chambers ^14^, and spatial memory tasks ^15^. Depending on the type of cognitive enrichment, beneficial effects on the animals’ well-being ^10^, postoperative cognitive dysfunction ^8^, motor function and neuropathology in a Huntington's Disease mouse model ^14^, and cognitive performance in an Alzheimer’s Disease mouse model ^15^ have already been found. These pieces of evidence suggest that enrichment facilitating manipulative and/or cognitive activities can foster the welfare of laboratory mice. However, some of the above-mentioned examples primarily facilitate manipulative or cognitive activities, while others involve aversive experiences, such as transferring the mice from their home environment to a testing arena. The latter could be seen as contradiction to the legal requirements for enrichment since laboratory animals “shall be given a degree of control and choice over their environment to reduce stress-induced behaviour” ^3^.

Therefore, we aimed to develop enrichment strategies for both manipulative and cognitive activities that can be presented to the mice in their home cages and used on a voluntary basis. For this, we were inspired by a modified concept of Thorndike’s puzzle box for cats ^16^. In Thorndike’s original experiments, cats were confined in a box and a palatable food reward was placed in front of the box. The cat had to engage with various mechanisms to open the box and reach the food ^16^. The original setup of Thorndike’s puzzle box had the potential to cause distress in the animals by being locked in the box. Modifications of this concept, where the food reward and not the animal is hidden in the box, and the animal must solve a series of mechanisms to access the reward, appear to better align with the criteria for an effective enrichment strategy. This ensures that the animal is not affected by being confined while still engaging in problem-solving tasks to obtain the reward. Such so-called lockboxes are mechanical puzzles comprising a range of mechanisms (e.g., screws, levers, bolts, doors) which obstruct one another and must be opened sequentially in the correct order to reveal a food reward ^17, 18^. Lockboxes can be constructed with varying degrees of complexity, comprising of one step to multiple steps. They were used to investigate sequential problem-solving in various species, e.g., cockatoos ^19^, pigeons ^20^, keas ^21– 23^, corvids ^24– 28^, and great apes ^29– 31^. Simple 1-step and 2-step tasks requiring to move/remove objects (e.g., a lid, a petri dish, paper, a window) were previously developed for wild house mice *(Mus musculus)* and striped field mice ( *Apodemus agrarius*) ^32, 33^. However, more sophisticated, and complex lockboxes suitable for mice have not been developed yet.

Against this background, our objective was to design lockboxes for mice and explore various aspects in three substudies. In the substudy presented in this article, we examined the impact of the lockbox enrichment (LE) on the animals’ phenotype and affective state, in comparison to mice kept under conventional housing (CH) or super-environmentally enriched housing conditions (SEE). Additionally, we assessed whether the LE affected data variability and reproducibility. In the remaining two sub-studies, not covered in this article, we delved into the problem-solving strategies employed by mice when tackling the lockboxes and their facial expressions. Moreover, we aimed to analyze the impact of the different types of enrichment on adult neurogenesis. The findings of our study will contribute to a better understanding of whether LE can serve as an effective refinement strategy and positively influence the animals' affective state. Additionally, these results will give researchers insights into whether LE may affect behavioral or physiological variables of interest in their studies, which need to be considered before introducing a novel type of enrichment.

## Methods

### Ethics statement

Research involving animals was performed according to the guidelines of the German Animal Welfare Act and the Directive 2010/63/EU for the protection of animals used for scientific purposes. Approval for animal experimentation and maintenance of the animals was granted by the Berlin State Authority (referred to as “Landesamt für Gesundheit und Soziales”, permit number: G0249/19). The pre-registration of the study can be found in the animal study registry (animalstudyregistry.org, doi: 10.17590/asr.0000237).

All efforts were made to ameliorate suffering of the mice: the mice were provided a large floor space, i.e., two Makrolon type III cages for 4 mice. A simulated sunrise smoothly increased the light intensity in the room before the lights turned on. Tunnels were used to handle the mice, which reduces stress and anxiety. The lock boxes were introduced as enrichment items.

### Animals, enrichment types, and handling method

A total of 36 female C57BL/6J mice, that were free of pathogens listed in the FELASA recommendations ^34^, were sourced from Charles River Laboratories (Sulzfeld, Germany). The animals were divided into three batches (i.e., 12 animals per batch) that were delivered at different time points. Each batch included four animals of each study group (three groups: LE, CH and SEE). The individual animals were considered as experimental units. The study planning included both sexes. Due to the time-consuming nature of the study, thus far, we have only been able to examine female animals. The estrus cycle phases were not assessed.

**Figure 1.**
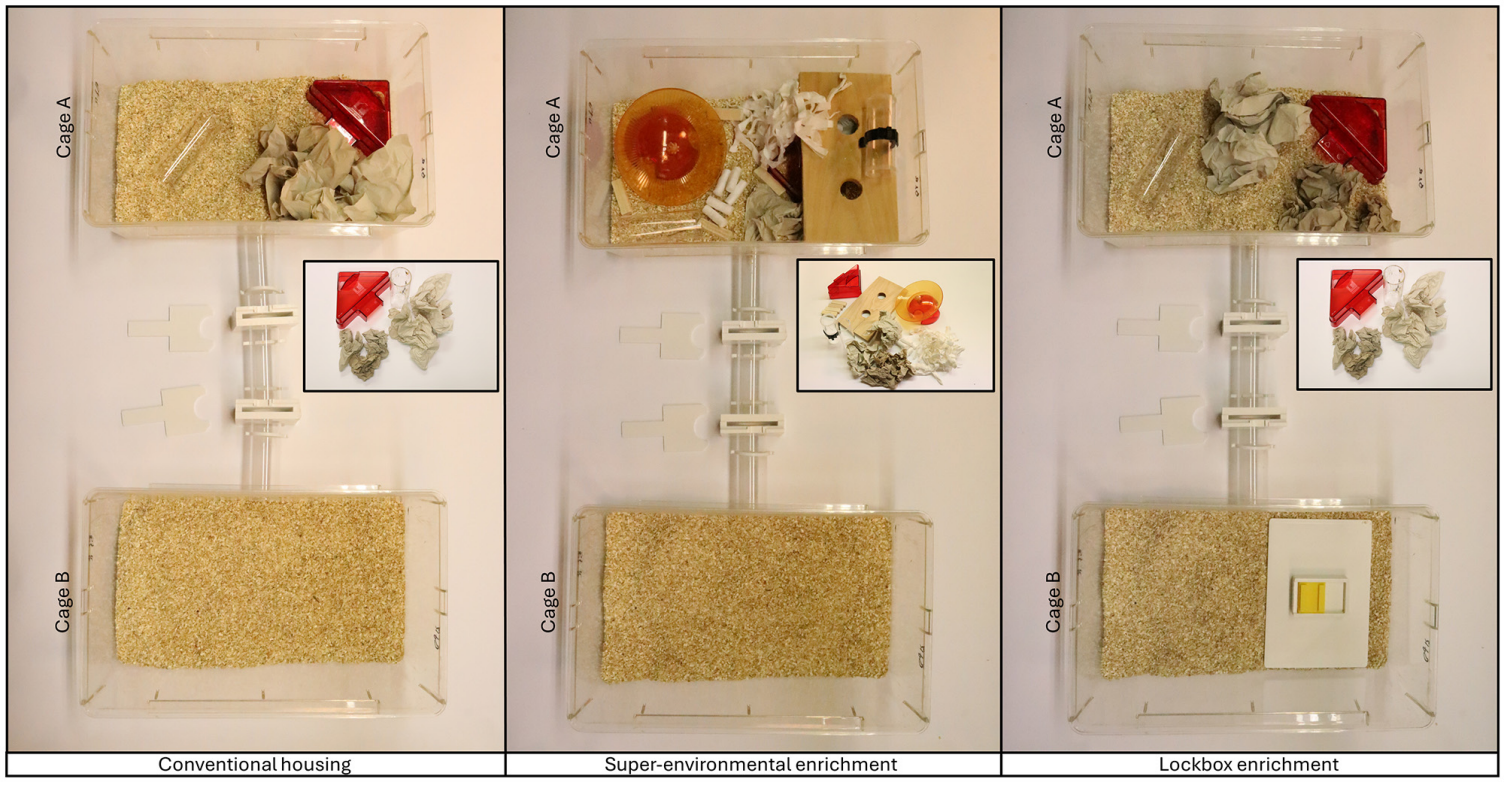
Home cage apparatuses of the study groups. The cage grid with food pellets and water bottle, as well as the filter tops were removed for the photographs. In the super-environmentally enriched cages, the tunnel attached to the cage grid with a clip was positioned on top of the wood board for photography purposes. The inserted images of the enrichment items illustrate the contents of cage A. The cages were connected with a gate comprising three transparent tubes with barriers and two door systems. The doors were placed next to the gate for the photographs and can be used to open or lock the gate. The only difference between the conventional housing and the lockbox enrichment group was the (temporary and permanent) access to the lockboxes in cage B.

Upon reaching approximately four weeks of age, the mice arrived at the animal facility and housed in filter top cages maintained under specifically pathogen free barrier conditions. The environmental conditions were set at a room temperature of 22 ± 1 °C, a relative humidity of 55 ± 10 %, and a 12/12-hour light/dark cycle. The light cycle began at 7 AM (summertime: 8 AM) and a wake-up light (Philips HF 3510, 100–240 vac, 50–60 Hz, Philips GmbH Market DACH, Hamburg, Germany) simulated the sunrise by gradually increasing light intensity starting at 6:30 AM (summertime: 7:30 AM). The mice were accommodated in groups of four (i.e., animals of the same study group were housed together) in two Makrolon type III cages (dimensions: 39 × 23 × 15 cm; designated as cage A and cage B), connected by a gate ( Figure 1). The gate comprised three transparent tubes (11.5 × 4 cm, custom-built from GEHR PMMA XT® ACRYL, Mannheim, Germany) with barriers (as described by Habedank *et al.* ^35, 36^), linked by two doors systems (custom-built, 3D printed; STL files can be found on Zenodo ^37^), that could be opened and locked. In both cages, ad libitum access to food pellets (LASvendi, LAS QCDiet, Rod 16, autoclavable) and tap water was provided. Fine wooden bedding material (JRS Lignocel FS14, spruce/ fir, 2,5-4 mm) was used for both cages.

**Figure 2.**
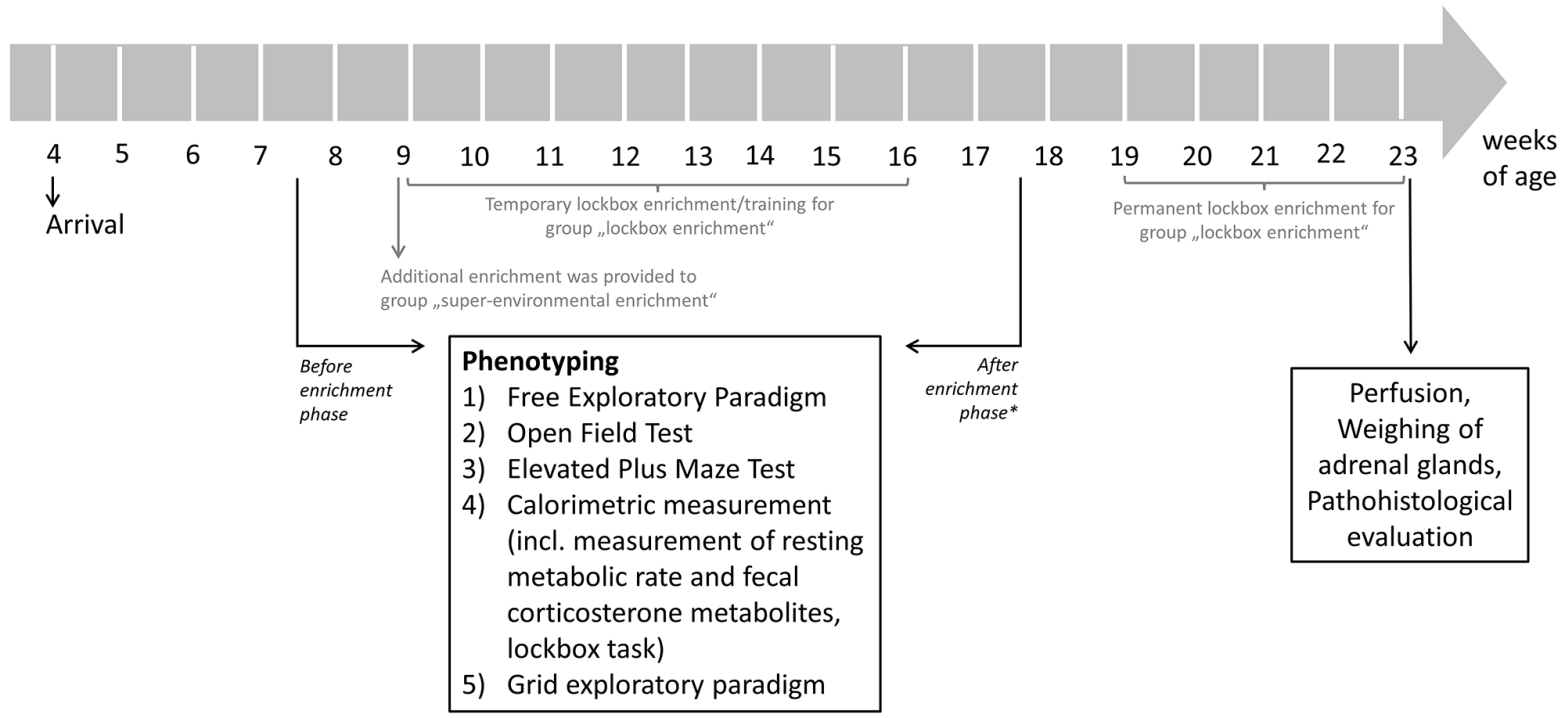
Flow chart of testing schedule. The phenotyping test battery was conducted before and after the 2-month enrichment phase. * While the name of the phase may suggest otherwise, it's important to note that the enrichment was not removed from either the lockbox enrichment group or the super-environmental enrichment group after the mice were tested for the second time.

The animals were assigned to three study groups by simple randomization: CH (n = 12), SEE (n = 12), and LE (n = 12). Figure 1 shows the home cage apparatuses of the three study groups. At the beginning of the study, cages of all study groups contained the same resources. Cage A was equipped with a red triangular plastic house with two exits (1 long side: 14.5, 2 short sides: 11.5, height: 5 cm, TheMouseHouse, Tecniplast), a transparent handling tunnel (length: 11.5, outside diameter: 4 cm, custom-built from GEHR PMMA XT® ACRYL,Mannheim, Germany), and three thin (cellulose, unbleached, layers, 20x20cm, Lohmann & Rauscher, Neuwied, Germany) as well as two thick (23 × 24,8cm folded, Essity ZZ Towel) paper tissues for nesting. In addition to this, both the SEE and LE groups were provided with further enrichment items after approximately five weeks ( Figure 2). In the SEE group, five cotton rolls (Gr.3, UNIGLOVES, Troisdorf, Germany) and 3–4 g white paper strips (LILLICO, Biotechnology Paper Wool) for nesting, a second transparent tunnel attached to the cage lid with a tunnel clip (polycarbonate, suitable for 38 mm (Dia) tunnels, Datesand, Bredbury, United Kingdom), a red mouse igloo with a yellow running plate (round house: 105 mm in diameter, 55 mm in height; round plate: 150 mm in diameter, ZOONLAB GmbH, Castrop-Rauxel, Germany), a second plane with two holes (dimensions: 23 × 12.5 × 1 cm; diameter of holes: 3 cm; 2 hole lying boards for cage type III, ABEDD, Kalnciems, Latvia), as well as four wooden gnawing blocks were placed into cage A. The LE group had temporary access to different types of lockboxes in cage B, which later became permanent, as described in detail in the section on lockbox training. In this group, the lid grid of cage B was replaced with transparent acrylic glass in an area of 22 × 15 cm. For the other two groups, cage B remained empty and the cage lid was not modified.

The animals in all study groups had unrestricted access to both cages, except for the LE group, whose access to cage B was unrestricted except for the short training periods conducted as part of the temporary LE, as described below in the section on lockbox training.

All animals were handled using a tunnel, i.e., the mice voluntarily entered the tunnel. In the first two weeks, they were habituated to the voluntary tunnel handling by the animal technicians and experimenters. For this, the protocol for voluntary tunnel handling, accessible at the Refinement Wiki of Norecopa, was shortened from 3 to 2 weeks ^38^.

### Animal identification

After acclimating to the animal facility for a week, radio frequency identification (RFID) transponders were subcutaneously implanted into the dorsocervical region using an application cannula (ISO-Compliant Transponder, Peddymark Limited, Redhill, Surrey, United Kingdom; cannula: 2.6 × 0.15 × 40 mm) during a brief isoflurane anesthesia (induction: 4 % isoflurane, maintenance: 1–2 % isoflurane; carrier gas: 100 % oxygen, flow: 1 liter/min; Isofluran CP 1ml/ml, CP-Pharma Handelsgesellschaft mbH, Burgdorf, Germany). In the evening before transponder implantation, 1 mg/kg meloxicam (Loxicom 0.5 mg/ml orale Suspension, Elanco Animal Health, Bad Homburg, Germany or Metacam 0.5 mg/ml Suspension zum Eingeben, Boehringer Ingelheim, Ingelheim am Rhein, Germany) was orally given. In addition to RFID transponders, color marking of the tail (edding 750 paint marker, edding International GmbH, Ahrensburg, Germany) was employed for visual identification of the individual mice.

### Testing schedule

Figure 2 demonstrates the testing schedule of the present study. Briefly, the mice arrived at the age of approximately four weeks at the animal facility and habituated to the new environment before the experiments started. Before the additional enrichment was provided the SEE and LE groups, a phenotyping test battery was conducted at the age of 7–8 weeks to measure baseline values of each animal. Until this point in time animals of all groups were housed under the same conditions (i.e., these conditions were equal to the CH). At the age of approximately 9 weeks, the additional environmental enrichment was provided to the SEE group and LE group was allowed temporary access to the lockboxes, which is further described in the section on lockbox training. After the 2-month enrichment phase, the phenotyping test battery was conducted for a second time to evaluate effects of the different types of enrichment. Subsequently, the animals of the LE group had permanent access to lockboxes (i.e., 24 hours per day). A lockbox baited with four oat flakes (kernel oat flakes, Wurzener Nahrungsmittel GmbH, Wurzen, Germany) was placed into cage B and was replaced once a day with another lockbox. The animals of the other study groups were kept under the same housing conditions as during the 2-month enrichment phase, i.e., the enrichment was not removed. Additionally, the same amount of oat flakes, as was filled in the lockboxes, was provided daily to cage B of the other study groups. An acoustic signal (i.e., careful, brief rattling on the gate system) was emitted to signal the animals that the oat flakes had been added to the cage. At the age of approximately 23 weeks, transcardiac perfusion was carried out, adrenal glands were weighted, and animal bodies were prepared for further pathohistological evaluation.

For another substudy, which is not further described in the present article, the brains were removed to examine the impact of the different types of enrichment on adult neurogenesis. As pretreatment for this examination, the animals were intraperitoneally injected with the proliferation marker chlorodeoxyuridine (CldU; 50 mg/kg; 10 μl/g bodyweight) before and the marker iododeoxyuridine (IdU; 50 mg/kg; 10 μl/g bodyweight) after the enrichment phase on three consecutive days. At both time points, the injections were carried out after the phenotyping test battery.

Originally, it was planned to measure home cage activity of the individual mice using the Mouse Position Surveillance System (MoPSS), an RFID based tracking system. However, due to technical malfunctions of the MoPSS devices in two batches, data could not be analyzed. These technical difficulties were solved in the updated version of the MoPSS published by Habedank *et al.* ^35^.

### Lockboxes

**Figure 3.**
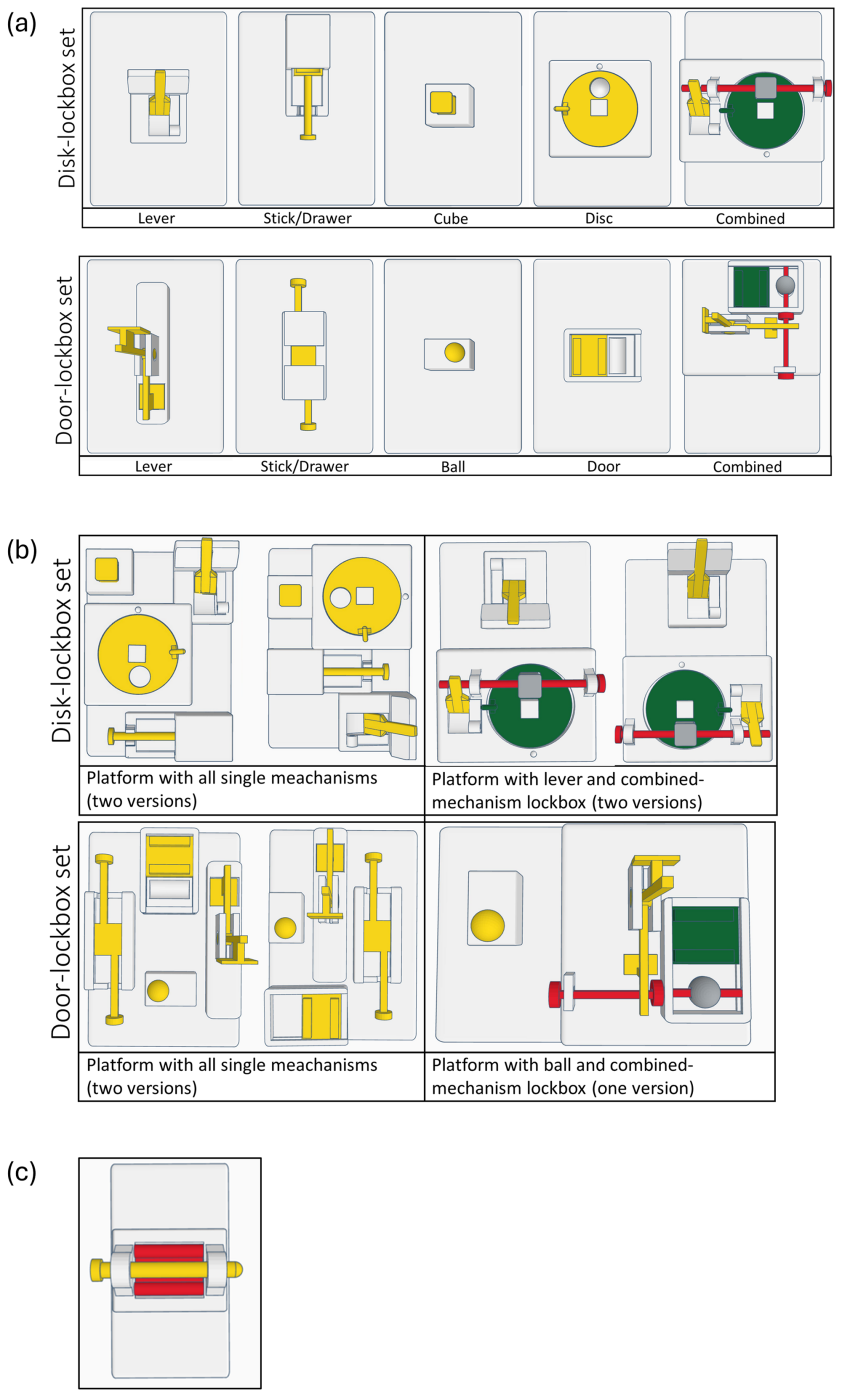
( **a**) Lockbox sets comprising of four single-mechanism lockboxes and a combined-mechanism lockbox. ( **b**) Multiple-lockbox platforms. For each lockbox set, two versions of a platform with all single mechanisms were designed, allowing to present all lockboxes simultaneously to the mice. For the door-lockbox set one version and for the disc lockbox set two versions of a platform with a single-mechanism and the combined-mechanism lockbox were created. The different versions allowed the mechanisms to be positioned at a different spot within the cage in each trial. ( **c**) 2-step lockbox presented to all mice at the end of the calorimetric measurement.

3D models of the lockboxes were designed using the computer-aided design software Tinkercad ( www.tinkercad.com). Models were transferred to 3D printable files using the slicing application Cura SteamEngine 4.4.0. The lockboxes were 3D printed using an Ultimaker 3 Extended and an Ultimaker S3 (0.4 mm nozzles). Polylactic acid (PLA) of different colors was used as 3D printing material (Filamentworld, Neu-Ulm, Germany: PLA filament white, yellow, red, grey; Formfutura, Nijmegen, The Netherlands: StoneFil PLA Filament Granite). The lockboxes consisted of a white platform and different mechanisms. Details on 3D printing and the STL files of the lockboxes can be found on Zenodo ^37^. Briefly, two sets of lockboxes were designed, with each set comprising four single-mechanism lockboxes (1 step) and one combined-mechanism lockbox (4 steps) ( Figure 3a). In the combined-mechanism lockbox, the four single mechanisms obstruct each other, requiring removal in the correct sequence to unlock the box.

Both lockbox sets comprised of similar mechanisms of slightly varying forms, i.e., a lever, a stick/drawer combination, a cube or ball, and a rotating disc or sliding door. To open the single-mechanism lockboxes, the mice had to move the lever to the other side, pull out the stick/drawer combination, remove the cube or ball, and finally move the disc or door. For solving the combined-mechanisms lockboxes, the mice had to open the mechanisms in the following order: lever, stick, cube, disc (disc-lockbox) or lever, stick, ball, door (door-lockbox). Short video sequences displaying the mice solving the lockboxes can be found on Zenodo ^37^.

To encourage the animals to engage with the lockboxes, they were baited with oat flakes as food reward (i.e., single-mechanism lockboxes were filled with half an oat flake, combined-mechanisms lockboxes with one oat flake). If an animal was not able to open the lockbox, it received the oat flakes after the lockbox training was completed for all mice.

### Lockbox training

On three consecutive days before the temporary lockbox training for the LE group began, eight oat flakes were added to cage B of all study groups to habituate them to the food reward. An acoustic signal (i.e., careful, brief rattling on the gate system) was emitted to signal the animals that the oat flakes had been added to the cage.

**Figure 4.**
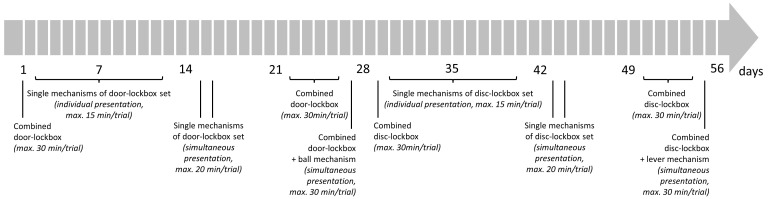
Lockbox training schedule. A trial was defined as the period in which the mouse was allowed the interact with a lockbox. It ended when the mouse opened the lockbox or when the maximum trial time expired.

The lockbox training started on the following day ( Figure 4). The mice were trained in the morning after the lights turned on. Each day, the order of the mice was randomized by drawing cards with the mouse ID on them. To begin the doors of the gate system were closed when all mice were in cage A and cage B was empty. A white screen was placed between cages A and B to prevent the mice from observing each other while solving the lockboxes. After a lockbox was placed into cage B, the first mouse was allowed to enter cage B and interact with the lockbox. For this the door closer to cage A was opened while the door closer to cage B remained locked. All mice usually attempted to access cage B and formed a queue inside the tube. When the mouse to be tested was the first animal in the queue, the door closer to cage A was closed and the door closer to cage B was opened. Once the mice entered cage B, this door was also locked. A trial, defined as the period in which the mouse was allowed to interact with a lockbox, ended when the mouse opened the lockbox or when the maximum trial time expired. The maximum trial time varied in the training phases and was between 15 and 30 minutes ( Figure 4). At the end of a trial, the experimenter waited until the mouse stopped interacting with the lockbox, before removing the lockbox from the cage. If the mouse continued interacting with it for more than one minute, the experimenter gently guided it away and removed the lockbox. After a mouse completed all planned trials of a day, it left cage B through the gate system. An acoustic signal (i.e., careful, brief rattling on the gate system) was emitted to signal the mice that the doors were opened, and the next mouse could enter cage B. There were four sets of each lockbox, i.e., a set for each mouse to prevent any odor cues. After the training, the lockboxes were cleaned with 70 % ethanol and left to air dry overnight.

Figure 4 demonstrates the sequence in which the lockboxes were presented to the mice. This sequence was first conducted for the disc-lockbox set starting on day 1, followed by the door-lockbox set beginning on day 29. Briefly, on day 1 (disc-lockbox set) or day 29 (door-lockbox set), the combined lockbox, in a locked state, was presented to the mice (1 trial/day). On day 2–12 (disc-lockbox set) or day 30–40 (door-lockbox set), the animals had access to the single mechanisms of the lockbox set. All four single mechanism lockboxes were presented once a day in a randomized order (1 trial/lockbox/day, i.e., 4 trials/day). Before the first trial of each single-mechanism lockbox on day 2 (disc-lockbox set) or day 30 (door-lockbox set), the animals were allowed to explore the baited lockboxes in an open state for a minute. Then it was rebaited and locked. On days 15 and 16 (disc-lockbox set) or days 43 and 44 (door-lockbox set), all four single-mechanism lockboxes were simultaneously presented to the mice twice (2 trials/day). There were versions of two platforms, each comprising all four single mechanisms, though the mechanisms were arranged differently on each platform ( Figure 3b). This allowed the four mechanisms to be located in a different spot within the cage for each trial. After that on days 22–26 (disc-lockbox set) or days 50–54 (door-lockbox set), the mice were trained on the combined lockbox. On day 22 (disc-lockbox set) or day 50 (door-lockbox set), the mice could explore the baited lockbox in an open state for a minute before it was rebaited and locked, and the trial began. While on day 22–25 (disc-lockbox set) or day 50–53 (door-lockbox set) 1 trial/day was conducted, 3 trials/day were performed on day 26 (disc-lockbox set) or day 54 (door-lockbox set). In the first trial, all mechanisms of the lockbox, in the second trial only the disc/door mechanism, and in the third trial only the cube/ball mechanism and disc/door mechanism were locked. On day 27 (disc-lockbox set) or day 55 (door-lockbox set), a platform comprising the combined-mechanism lockbox and a single-mechanism lockbox was presented to the mice twice (2 trials/day). In the second trial, the two lockboxes were rearranged by either rotating the platform by 180 degrees (door-lockbox) or by using the second version of this platform (disc-lockbox).

During the lockbox training, the mice were video-recorded using three Basler Cameras acA1920-40um (Lens LM25HC7, f = 25mm, k = 1.4, Kowa, Nagoya, Japan3) and two Intel Real Sense cameras. The video material was used in other substudies to analyze the problem-solving strategies of the mice and their facial expression when solving the lock boxes.

### Free Exploratory Paradigm

The Free Exploratory Paradigm was conducted to investigate trait anxiety-related behavior. To enable a repeatability analysis, the Free Exploratory Paradigm included a habituation phase and was conducted over three consecutive days. There were four testing apparatuses for the Free Exploratory Paradigm allowing to simultaneously test the four animals of a cage group, i.e., a test apparatus for each mouse. A testing apparatus consisted of a starting cage (Makrolon type III cage). A handful of used bedding was scattered on top of the clean bedding. Moreover, the cages were equipped with a red triangular plastic house, two thin paper tissues, and a thick paper tissue. Food pellets and tap water were provided in the gridded cage lid. The cage was connected via two tunnels (length: 11.5, outside diameter 4 cm, custom-built from GEHR PMMA XT® ACRYL,Mannheim, Germany) to an arena (50 × 50 cm, height of walls: 50 cm). The two tunnels were linked by a door system (custom-built, 3D printed, STL files can be found on Zenodo ^37^) denying access to the arena. The illumination level was set to 90 ± 9 lx in the center and the corners (mean ± standard deviation). After all animals were individually transferred with the tunnel from their group cage to the respective starting cage, the doors remained closed for a minute and were then opened, allowing the mice to voluntarily enter the arena. Every day the Free Exploratory Paradigm was conducted, the mice were allocated to a different testing apparatus from the four available. The 25-minute testing period was recorded from the top with a Basler acA1920-40um camera (Lens LM8HC7, f = 8mm, k = 1.4, Kowa, Nagoya, Japan; 41 monochrome frames per second) using the Basler Video Recording Software ( www.baslerweb.com/en/software/video-recording). After 25 minutes had elapsed, the mice were transferred with the tunnel to the group cage. The testing arena was clean with 70 % ethanol at least 12 hours before the test was performed. The experimenter manually counted the number of fecal boli and urinary spots. Additionally, variables of interest were the number of mice entering the arena, the latency to enter the arena, the distance travelled in the arena, and the percentage of time moving in the center or periphery relative to the time moving in both zones.

The number of mice entering the arena and the latency to enter the arena were manually assessed from the videos. A mouse entered the arena when all four paws left the tunnel and were placed on the floor of the arena. The distance travelled in the arena, and the time moving in the center or periphery were automatically analyzed from the videos using DeepLabCut (version 2.3.4, https://github.com/DeepLabCut/DeepLabCut) ^39, 40^ and the DLCAnalyzer ^41^ ( https://github.com/ETHZ-INS/DLCAnalyzer), as described below.

### Open Field Test

The Open Field Test was performed to examine state anxiety-related behavior. To enable a repeatability analysis, the Open Field Test included a habituation phase and was conducted over three consecutive days. Every day, the order in which the mice were tested was altered. The animals were individually transferred with the tunnel from their group cage to the testing arena (80 × 80 cm, height of walls: approximately 60 cm, material: polyvinyl chloride). The illumination level was set to 104 ± 3 lux in the center and 59 lux ± 3 in the corners (mean ± standard deviation). The tunnel was attached to the hole (diameter: 4 cm) in the wall of the testing arena and the mouse climbed from the tunnel into the starting zone, which was located in a corner and separated from the rest of the arena by a starting cylinder (15 × 15 cm). Then a plug was used to close the hole in the wall. After a 1-minute period, the starting cylinder was removed, allowing the mouse to freely explore the arena for 10 minutes. The 10-minute testing period was recorded from the top with a Basler acA1920-40um camera (Lens LM8HC7, f = 8mm, k = 1.4, Kowa, Nagoya, Japan; 41 monochrome frames per second) using the Basler Video Recording Software ( www.baslerweb.com/en/software/video-recording). After 10 minutes had elapsed, the mouse was transferred with the tunnel to the group cage and the testing arena was cleaned with 70 % ethanol. The experimenter manually counted the number of fecal boli and urinary spots. Additionally, variables of interest were the time spent in the central zone, time spent in the peripheral zone, and distance moved, which were automatically analyzed from the videos using DeepLabCut (version 2.3.4, https://github.com/DeepLabCut/DeepLabCut) ^39, 40^ and the DLCAnalyzer ^41^ ( https://github.com/ETHZ-INS/DLCAnalyzer), as described below.

### Elevated Plus Maze Test

The Elevated Plus Maze Test was carried out to measure state anxiety-related behavior. To enable a repeatability analysis, the Elevated Plus Maze Test included a habituation phase and was conducted over three consecutive days. Every day, the order in which the mice were tested was altered. The testing apparatus consisted of two open and two closed arms (dimensions of the arms: 30 × 5 cm, center: 5 × 5 cm, material: polyvinyl chloride). The open arms without side walls were perpendicular to the closed arms with 20-cm-high side walls. The testing apparatus was elevated to a height of 50 cm above the floor. The open arms were surrounded by a raised ledge (6 mm thick, 6 mm high). The illumination level was set to 92 ± 3 lx in the center, 147 ± 3 lux on the open arms, and 31 ± 6 lux on the closed arms (mean ± standard deviation). The animals were individually transferred with the tunnel from their group cage to the center of the testing apparatus, facing a closed arm and away from the experimenter. The test started when the mouse left the tunnel with all four paws. The 5-minute testing period was recorded from the top with a Basler acA1920-40um camera (Lens LM8HC7, f = 8mm, k = 1.4, Kowa, Nagoya, Japan; 41 monochrome frames per second) using the Basler Video Recording Software ( www.baslerweb.com/en/software/video-recording). After 5 minutes had elapsed, the mouse was transferred with the tunnel to the cage and the testing arena was cleaned with 70 % ethanol. The experimenter manually assessed defecation the number of fecal boli and urinary spots. Moreover, variables of interest were the distance travelled, the time spent on the open or closed arms, the number of transitions to the open or closed arms, the ratio of the time spent on the opens arms to the time spent on the closed arms, and the number of protected stretch attend. It must be noted that the center of the Elevated Plus Maze was neither part of the open nor the closed arms, i.e., the time spent in the center of the Elevated Plus Maze Test was not analyzed. DeepLabCut (version 2.3.4, https://github.com/DeepLabCut/DeepLabCut) ^39, 40^ and the DLCAnalyzer ^41^ ( https://github.com/ETHZ-INS/DLCAnalyzer) were used to automatically analyze the distance travelled, the time spent on the open or closed arms, and the number of transitions to the open or closed arms from the videos, as described below. The number of protected stretch attend was manually assessed using BORIS ^42^.

### Videos analysis of Free Exploratory Paradigm, Open Field Test, and Elevated Plus Maze Test

DeepLabCut (version 2.3.4, https://github.com/DeepLabCut/DeepLabCut) was used for body part tracking ^39, 40^. For the Free Exploratory Paradigm and Open Field Test, the four corners of the testing arena and the body center were labeled. For the Elevated Plus Maze Test, the four corners of the center, the two corners of each arm, and the body center were labeled. The body center was defined as the half-length between the nose and the tail base. For training 30 frames were taken from 10 videos (5 % was used for validation). We used a ResNet50-based neural network ^43, 44^ and trained for 500,000 training iterations (Free Exploratory Paradigm), for 900,000 training iterations (Open Field Test), or 100,000 training iterations (Elevated Plus Maze Test) with otherwise default parameters and validated with one shuffle, which resulted for the Free Exploratory Paradigm in a test error of 2.85 pixels and a train error 3.51 pixels (image size was 1920 by 1080), for the Open Field Test in a test error of 4.49 pixels and a train error 4.28 pixels (image size was 1936 by 1216), and for the Elevated Plus Maze Test in a test error of 2.78 pixels and a train error 3.06 pixels (image size was 1936 by 1216). A p-cutoff of 0.6 was chosen. These networks were then used to analyze videos from all mice tested in the respective tests. The csv files generated in DeepLabCut were further processed with the DLCAnalyzer (i.e., R code for OFT analysis of multiple files for the Free Exploratory Paradigm and Open Field Test, R code for EPM analysis of multiple files for the Elevated Plus Maze Test, https://github.com/ETHZ-INS/DLCAnalyzer) ^41^ using R (version 4.0.3; www.r-project.org).

### Measurement of resting metabolic rate in a calorimetry system

As described previously, the indirect calorimetry was employed to analyze the resting metabolic rate of the mice ^7^. Briefly, air was perfused through the four measurement cages of the calorimetry system (TSE PhenoMaster, TSE Systems GmbH, Bad Homburg, Germany) and an empty reference cage. During the measuring period, oxygen levels in the measurement cage were reduced, while carbon dioxide levels increased due to the animals' respiration. After the air flowed through both cages, the air composition in the measurement cages and reference cage was compared. The metabolic rate of the animals was determined by calculating the difference in air compositions. The measurement for each cage lasted 2 min, meaning a measuring cycle of five cages lasted 10 minutes and 6 data points were collected for each of the four animals within an hour.

Since the calorimetry system was located in another room (same light cycle as in the housing room), the mice were transported in group cages (Makrolon type III cages; food pellets and water, bedding, nesting material, and environmental enrichment as described in the section on “animals, enrichment types, and handling methods”) the day before the measurement was started to habituate the animals to the room. In the morning of the next day, the mice were weighted and then individually transferred using the tunnel from their group cage to the measurement cages of the calorimetry system (dimensions at floor level: approximately 15 × 30 cm, dimensions at top level: 17.5 × 35.5 cm, height: 13 cm; floor covered with fine wooden bedding material, JRS Lignocel FS14, spruce/ fir, 2,5-4 mm). After all mice had been transferred to the calorimetry system, the measurement was started. In the morning of the next day, after the lights turned on, the 2-step lockbox ( Figure 3c) was presented to the mice, as described in the section on “lockbox task in the calorimetric apparatus”. Subsequently, the calorimetric measurement was stopped, and the Grid Exploratory Paradigm was conducted. Before the mice were transferred with the tunnel to their group cages and transported to their housing room, the mice were weighted.

Variable of interest was the resting metabolic rate (RMR), which was quantified as the oxygen consumption rate (VO2) during the animals' resting phases. For the RMR analysis, the data were standardized by excluding the active phases of the animals, ensuring the same number of data points for each individual was analyzed. As only four animals could be tested simultaneously in the calorimetry system, the starting time slightly varied between groups. The starting time of the group with the latest measurement start was used as a reference point (11:13 AM). The first hour of recording (i.e., six data points) was omitted due to increased activity during habituation. Additionally, data points after 7:00 AM on the following day were excluded, as the onset of lights increased activity once again and the 2-step lockbox was presented to the mice. This resulted in 113 data points per individual for RMR analysis. To distinguish resting phases from active phases, a plot of cumulative frequency percentage against the measured VO2 was generated. Through segmented linear regression, the threshold between the VO2 levels of the resting and active phases was calculated. Data points falling below this threshold were utilized for determining the RMR (R package 'segmented'; R version 4.2.2; www.r-project.org) ^45^.

### Lockbox task in the calorimetric apparatus

After the animals spent a night in the measurement cages of the calorimetric system, a 2-step lockbox was presented to them ( Figure 3c). The 2-step lockbox comprised a stick that needed to be pulled out first before a block could be removed, unveiling the food reward (i.e., the oat flake). In the morning, after the lights turned on at approximately 7 A.M., the experimenter carefully opened the cages one after the other and, with the gloved hand, shifted bedding material from the front part of each cage to the rear. This was conducted within a 10-min measuring cycle and was then repeated in a second measuring cycle. Subsequently, the open 2-step lockbox was filled with an oat flake and was placed in the front of the cage for a 10-min measuring cycle. Then the lockbox was rebaited with an oat flake and locked. The animals were allowed to explore and interact with the lockbox on a voluntary basis for approximately 30 minutes. This process was considered as habituation. After 30 minutes, the lockbox was rebaited with another oat flake if it was open; otherwise, the experimenter’s hand only touched the lockbox to ensure a consistent procedure for all animals, regardless of whether they opened it or not. After the enrichment phase, the 30-minute phase, during which the baited lockbox was presented in a locked state, was eliminated from the habituation period because the animals were already familiar with the lockbox at this timepoint. The animals were recorded with a Basler acA1920-40um camera (Lens LM25HC7, f = 25mm, k = 1.4, Kowa, Nagoya, Japan; 30 monochrome frames per second) and behavioral analysis of the (last) lockbox trial before and after the enrichment phase were manually performed using BORIS ^42^.

Variables of interest were the interaction time with the lockbox and the time point when it was opened. Moreover, resting phases were observed. These variables were defined, as follows: interactions with both non-removeable and removeable lockbox elements included sniffing, touching with paws or nose, climbing, gnawing, digging, eating or carrying around lock box elements. The lockbox was deemed open when the mouse had removed both stick and the block, allowing access to the oat flake. Resting behavior was evident when a mouse was lying curled up on its side or sitting in a curled-up position and the face tucked into the body ^46^. During these resting phases, a mouse remained still, but occasional brief single twitches could be observed ^46^. Another variable of interest was the consumption rate (VO2) during lockbox trials. The three values of the oxygen consumption rate measured during the last lockbox trial were averaged for the time point before and after the enrichment phase, respectively. Only mice that interacted with the lockboxes and did not rest were included in the analysis.

### Measurement of fecal corticosterone metabolites

All fecal pellets that were excreted when mice were kept for approximately 24 hours in the measuring cages of the calorimetric system were collected using forceps. The samples were frozen at −80°C. Extraction and analysis of fecal corticosterone metabolites (FCMs, i.e., 5α–3β, 11β–corticosterone metabolites in ng/0.05 g feces) were conducted later according to previously established methods ^47– 49^.

### Grid Exploratory Paradigm

Grid Exploratory Paradigm was performed to examine trait anxiety-related behavior. In the literature, this test is also called the “Free Exploratory Paradigm” ^50^ but to avoid confusion with the "Free exploration Paradigm" below, we named it more precisely here. The Grid Exploratory Paradigm was conducted after the calorimetric measurement was completed. The cage lid was removed, and the cage was moved to a rack on the opposite side of the room. Then a ladder (30.5 × 11 cm), constructed from metal grid and a frame (custom-built, 3D printed), was placed into the cage (i.e., start of the test) and the mice were allowed to explore the ladder for 10 minutes. The test was repeated three times with a break of 5 minutes between the trials. The ladders were cleaned with 70 % ethanol after each trial.

The animals were recorded with a Basler acA1920-40um camera (Lens LM25HC7, f = 25mm, k = 1.4, Kowa, Nagoya, Japan; 30 monochrome frames per second). Variables of interest were the latency to explore, the total time of exploration, and the number of explorations. Exploration was defined as all four paws having contact with the ladder or the cage edge, except from rearing events or when the mouse was lifting a leg while walking. Behavioral analysis were manually performed using BORIS ^42^.

### Bodyweight

Mice were weighted weekly during the general health inspection and cage change. For this, they voluntarily entered the tunnel and then climbed from the tunnel onto the scale. After weighing, they voluntarily climbed from the scale into the tunnel and were transferred to their home cage.

### Perfusion

At the end of the study, transcardiac perfusion was conducted using the Perfusion ONE System (Leica Biosystems, Nussloch, Germany). To induce anesthesia, a mixture of ketamine and xylazine was administered intraperitoneally at a volume of 10 μL/g bodyweight was administered intraperitoneally using 30 Gauge needles. The initial planned dosage of 80 mg/kg ketamine (Ketabel 100 mg/ml Injektionslösung, bela-pharm, Vechta, Germany) and 16 mg/kg xylazine (Xylazin, 20 mg/ml Injektionslösung, WDT, Garbsen, Germany) had to be adjusted to 180 mg/kg ketamine and 20 mg/kg xylazine since the mice did not reach surgical tolerance. Following the intraperitoneal injection, the loss of the righting reflex, lid reflex of both eyes, and pedal withdrawal reflex of all limbs were assessed. If the lid reflex on one side or pedal withdrawal of at least one limb persisted 15 min after anesthesia induction, an additional dose of ketamine and xylazine was administered intraperitoneally. Once all reflexes were lost, the thorax was opened below the sternum and the heart was dissected free to insert the cannula into the left ventricle. The perfusion pressure was set at 150 mmHg and the right atrium of the heart was opened to drain the perfusion fluid. Animals were perfused with 0.9% physiological saline solution (NaCl) for two minutes to wash out all blood from the vasculature, followed by perfusion with 4% paraformaldehyde. After perfusion, the adrenal glands were carefully removed, freed from adipose fatty tissue under an incident light microscope, and weighted with a precision scale. For further pathological examinations, the animal bodies were stored in a 4 % paraformaldehyde solution.

### Pathohistological evaluation

Samples of formalin fixed pars non-glandularis and glandularis of the stomach, small intestine, caecum, colon, mesenteric lymph node, liver, and kidney were paraffin embedded and cut into 3-µm sections followed by routine haematoxylin and eosin staining for pathohistological evaluation.

### Method of blinding

Computer-generated random numbers were used to assign animal IDs to a study group (i.e., the IDs did not contain any information on the study group). The persons analyzing the videos and conducting pathological examinations were blinded. When statistical analysis was carried out, the groups were named after numbers and numbers were replaced with the type of enrichment after the analysis was completed. Due to the obvious differences in the enrichment between the study groups, the animal care takers and experimenters could not be blinded when they handled the animals.

### Method of randomization

At arrival animals are assigned to a study group by simple randomization (i.e., the first mouse caught was assigned to the CH group, the second mouse to the SEE group, the third mouse to the LE group, etc.). For the phenotyping test battery, the order of the mice was randomized. Moreover, in the LE group, the order of mice was randomized every day. When the mice were trained on the single-mechanism lockboxes, the order of the lockboxes was randomized every day. The lock boxes consisting of several single mechanisms or both a single mechanism and a combined lockbox were presented in way that the location of the different lockboxes varied in each trial.

### Sample size calculation

The present study was exploratory. After three batches (i.e., three cages with four animals) per study group (i.e., CH, SEE, LE) were examined, the data were analyzed and effect sizes were determined. It was defined during the study planning that the fourth batch should not be examined if the data show an effect size = 0 or a relevant, statistically significant effect (p < 0.05) and a large effect size (e.g. for the difference between more than two means Eta-squared > 0.14, for the difference between two mean Cohens d > 0.8). There was a large effect in the total time of exploration (Eta-squared ≈ 0.18, p < 0.05) and the latency to explore (Eta-squared ≈ 0.15, p < 0.05) in the grid exploratory paradigm as well as in the number of mice solving a 2-step lockbox in the calorimetric apparatus (Cramer-V ≈ 0.5). Therefore, the fourth batch was not investigated in order to reduce the number of animals used for the experiment.

## Statistical analysis

Statistical analysis was carried out using IBM SPSS Version 26 (IBM Corporation, Armonk, NY, USA, https://www.ibm.com/products/spss-statistics) and the open-source statistical software R (version 4.2.2, www.r-project.org) ^51^. For data analysis using R, the R packages lme4 ^52^ and lmerTest ^53^ were utilized to fit linear mixed-effects models (LMMs) of varying complexity. Animal (i.e., the individual mice, n = 36; referred to as MouseID in the data) and cage (i.e., groups of four animals, 9 levels) were treated as random factors in all models (exceptions are described below). Fixed factors included group (i.e., the three different types of enrichment, 3 levels) and time (i.e., before and after the 2-months enrichment phase, 2 levels) in the simpler model, or group, time, and interaction between group and time in the more complex model. Diagnostic plots (Q-Q plots, residuals versus fitted values, and histogram of residuals) were generated using the R packages ggplot2 ^54^, ggsci ^55^, and ggpubr ^56^ to assess the assumptions of the models and check for any deviations from normality or homoscedasticity. Post-hoc pairwise comparisons were performed using the R package emmeans ^57^ to evaluate differences between levels of the fixed effects. The false discovery rate (FDR) method was applied to correct p values. Model fits were compared using ANOVA to determine whether the additional complexity in the models resulted in a significant improvement in fit compared to the simpler models. The model selection was based on p, AIC, and BIC values.

If normality or homoscedasticity was violated, data were transformed. If data transformation failed, the data distribution was identified and, if possible, generalized linear mixed-effects models (GLMMs) were employed. Model fit comparisons were conducted as described above. For GLMMs, post-hoc pairwise comparisons were performed using the R package car ^58^. Alternatively, other non-parametric tests were applied.

Details on the model statistics are listed below for each parameter. Significant differences revealed by post-hoc pairwise comparisons of the interaction effect are shown in the figures and tables.

As a measure of relative variability, the coefficient of variation of 23 continuous variables was calculated for each study group by the following formula: (standard deviation/mean) × 100. Values before and after the enrichment phase were compared within each group to detect an increase or decrease.

### Grid Exploratory Paradigm

The Grid Exploratory Paradigm was carried out three times in a row. The explorative behavior seemed to increase over the trials and some animals tried to climb out of the cage in trial 2 and trial 3 which had to be prevented by the experimenter. Due to this disturbance of the animal’s behavior in these two trials, only trial 1 was used for further analysis.

Since the videos of the Grid Exploratory Paradigm were manually annotated, consistency of the data was determined by analyzing the interrater reliability between the person who labeled all videos and another observer who was asked to label six videos. The interrater reliability was calculated using BORIS ^42^ (Cohens Kappa) and interpreted according to Landis and Koch ^59^.

**Total time of exploration:** A LMM (fixed factors: group, time, interaction between group and time; random factors: animal, cage) was used to analyze the total time of exploration. Comparisons of model fits showed a significant difference in overall fit statistics between the models with and without the interaction between group and time. AIC and BIC values revealed that the complex model provided a better fit to the data.

**Latency to explore:** A GLMM with a Gamma distribution (fixed factors: group, time, interaction between group and time; random factor: animal) was employed to analyze the latency to explore. This decision followed the fitting of a LMM to the data, inspection of diagnostic plots, and identification of the data distribution using a histogram of the latency to explore. Given the warning regarding singularity in the GLMM with animal and cage as random factors, we opted to proceed with a simpler model that included only the animal as a random factor. Comparisons of model fits showed a significant difference in overall fit statistics between the models with and without the interaction between group and time, with the model with the interaction having lower AIC as well as BIC values. Since the complex model provided a better fit to the data, this model was chosen for analysis.

**Number of explorations:** A LMM (fixed factors: group, time; random factors: animal, cage) was used to analyze the number of explorations. Comparisons of model fits showed a significant difference in overall fit statistics between the models with and without the interaction between group and time. While the simpler model had a slightly lower BIC, indicating a preference for parsimony, the more complex model with the interaction term had a slightly lower AIC, suggesting a better overall fit to the data. This suggested that the more complex model provided a better balance between model fit and complexity. We advocated for the use of the more complex model as it offered a more comprehensive and interpretable analysis of the data.

### Free Exploratory Paradigm

**Distance travelled during habituation:** The Free exploratory paradigm was carried out on three consecutive days. Differences in the distance travelled between the days were analyzed using the Friedman test with Dunn-Bonferroni post-hoc test, as described previously ^60^. As a measure of relative variability of the distance travelled on the three days, the coefficient of variation was calculated. Due to a technical malfunction of the recording from the LE group on day 1 after the enrichment phase, four mice of this group had to be excluded from this analysis. A repeatability analysis was carried out using “rptR” library for RStudio to determine the source of the variance within the data ^61, 62^, as described previously by Rudeck *et al.* ^60^. Briefly, random factors were animal, group, or batch; the fixed factor was the distance travelled. The 95 % confidence interval was based on 500 bootstrap runs and 100 permutations ^60^. The likelihood ratio test was used to test for statistical significance of the repeatability of the distance travelled between the three days ^60^. The most important random factor was determined by calculating the repeatability “R” over the three days for all fixed factors including animal, group, and batch as multiple random grouping factors ^60^.

**Comparison of test-related variables across groups:** Differences between the groups were analyzed using the mean of the three days. Due to a technical malfunction of the recording from the LE group on day 1 after the enrichment phase, only data of day 2 and day 3 were averaged for these four mice. After the sum of the time moving in the center and in the periphery was calculated, the percentage of time moving in each location was determined. If the DLCAnalyzer did not detect a mouse moving in the center or in the periphery on all three days before or after the enrichment phase (i.e., the sum of the time moving in the center and in the periphery was equal 0), the mouse was excluded from the analysis of this variable. It must be noted that the number of mice moving in the arena detected by the DLCAnalyzer may vary from the number of mice that entered the arena with all four paws since we did not track the paws but only the body center using DLC.

**Distance travelled in the arena:** A LMM (fixed factors: group, time, interaction between group and time; random factor: animal) was used to analyze the distance travelled. We encountered convergence issues with the LMM including both animal and cage as random factors. After careful consideration, we decided to proceed with a simpler model including only animal as random factor. Although comparison of model fits revealed no significant difference in overall fit statistics between the models with and without the interaction between group and time, the inclusion of the interaction allowed for a more nuanced examination of the differential effects of the different groups across time. While the simpler model had a slightly lower BIC, indicating a preference for parsimony, the more complex model with the interaction term had a slightly lower AIC, suggesting a better overall fit to the data. This suggested that the more complex model provided a better balance between model fit and complexity. We advocated for the use of the more complex model as it offered a more comprehensive and interpretable analysis of the data.

**Percentage of time moving in the center or periphery:** LMMs (fixed factors: group, time; random factors: animal, cage) were used to analyze the percentage of time moving in the center or periphery. Comparisons of model fits showed no significant difference in overall fit statistics between the models with and without the interaction between group and time, with the models without interaction having lower AIC and BIC values. Since the simpler models provided a better fit to the data, they were employed for analysis.

**Number of mice entering the arena:** A GLMM with a binomial distribution (fixed factors: group, time; random factor: animal) was employed to analyze whether the mice entered the arena on at least one out of the three days the test was performed. Given the warning regarding singularity (GLMM excluding the interaction between group and time as fixed factor) and convergence (GLMM including interaction between group and time as fixed factor) issues in the GLMM with animal and cage as random factors, we opted to proceed with a simpler model that included only animal as a random factor. However, a warning regarding convergence issues still occurred for the models including the interaction as fixed factor. After careful considerations, we decided to proceed with the simpler including only time and group as fixed factors and animal as random factor.

**Latency to enter the arena:** A LMM (fixed factors: group, time, interaction between group and time; random factors: animal, cage) was used to analyze the latency to enter the arena. Comparisons of model fits showed a significant difference in overall fit statistics between the models with and without the interaction between group and time. AIC and BIC values revealed that the complex model provided a better fit to the data.

### Open Field Test

**Distance travelled during habituation:** The Open field test was carried out on three consecutive days. Differences in the distance travelled between the days were analyzed using the Friedman test with Dunn-Bonferroni post-hoc test. As a measure of relative variability of the distance travelled on the three days, the coefficient of variation was calculated. A repeatability analysis was carried out, as described above.

**Comparison of test-related variables across groups:** Differences between the groups were analyzed using the mean of the three days.

**Distance travelled and time spent in the center:** LMMs (fixed factors: group, time; random factors: animal, cage) was used to analyze the distance travelled and the time spent in the center. Comparisons of model fits showed no significant difference in overall fit statistics between the models with and without the interaction between group and time, with the models without the interaction having lower AIC as well as BIC values. The simpler models were chosen for analysis since they provided a better fit to the data.

**Time spent in the periphery:** A LMM (fixed factors: group, time, interaction between group and time; random factors: animal, cage) were used to analyze the time spent in the periphery. Comparisons of model fits showed a significant difference in overall fit statistics between the models with and without the interaction between group and time, with the models with the interaction having lower AIC as well as BIC values. The complex model was chosen for analysis since it provided a better fit to the data.

**Urination:** A GLMM with a binomial distribution (fixed factors: group, time; random factor: animal) was employed to analyze urination during the Open Field Test. Given the warning regarding singularity in the GLMM with animal and cage as random factors, we opted to proceed with a simpler model that included only the animal as a random factors. Comparisons of model fits showed no significant difference in overall fit statistics between the models with and without the interaction between group and time, with the model without interaction having lower AIC as well as BIC values. The simpler model was chosen for analysis since it provided a better fit to the data.

**Defecation:** A GLMM with a Poisson distribution (fixed factors: group, time; random factor: animal) was employed to analyze the number of fecal boli excreted during the Open Field Test. This decision followed the fitting of a LMM to the data, inspection of diagnostic plots, and identification of the data distribution using a histogram of the number of fecal boli. Given the warning regarding singularity in the GLMM with animal and cage as random factors, we opted to proceed with a simpler model that included only the animal as a random factor. Comparisons of model fits showed no significant difference in overall fit statistics between the models with and without the interaction between group and time, with the model without the interaction having lower AIC as well as BIC values. The simpler model was chosen for analysis since it provided a better fit to the data.

### Elevated Plus Maze Test

**Distance travelled during habituation:** The Elevated plus maze test was carried out on three consecutive days. Differences between the days were analyzed using the Friedman test with Dunn-Bonferroni post-hoc test. As a measure of relative variability of the distance travelled on the three days, the coefficient of variation was calculated. A repeatability analysis was carried out, as described above.

**Comparison of test-related variables across groups:** Differences between the groups were analyzed using the mean of the three days.

**Distance travelled:** A LMM (fixed factors: group, time; random factors: animal, cage) was used to analyze the distance travelled. Comparisons of model fits showed no significant difference in overall fit statistics between the models with and without the interaction between group and time, with the models without the interaction having lower AIC as well as BIC values. The simpler model was chosen for analysis since it provided a better fit to the data.

**Number of protected stretch attend:** A LMM (fixed factors: group, time; random factor: animal) was employed to analyze the number of protected stretch attend. Given the warning regarding singularity in the LMM with animal and cage as random factor, we opted to proceed with a simpler model that included only the animal as a random factor. Comparisons of model fits showed no significant difference in overall fit statistics between the models with and without the interaction between group and time. The model without interaction had lower AIC as well as BIC values. The simpler model was chosen for analysis since it provided a better fit to the data.

**Time spent on open arms or closed arms:** LMMs (fixed factors: group, time; random factors: animal, cage) were used to analyze the time spent on the open or closed arms. Comparisons of model fits showed no significant difference in overall fit statistics between the models with and without the interaction between group and time, with the models without the interaction having lower AIC as well as BIC values. The simpler models were chosen for analysis since they provided a better fit to the data.

**Number of transitions to open arms:** A GLMM with a Poisson distribution (fixed factors: group, time; random factors: animal, cage) was employed to analyze the number of transitions to the open arms. This decision followed the fitting of a LMM to the data, inspection of diagnostic plots, and identification of the data distribution using a histogram of the number of transitions to the open arms. Comparisons of model fits showed no significant difference in overall fit statistics between the models with and without the interaction between group and time, with the model without the interaction having lower AIC as well as BIC values. The simpler model was chosen for analysis since it provided a better fit to the data.

**Number of transitions to closed arms:** A LMM (fixed factors: group, time; random factor: animal) was used to analyze the number of transitions to the closed arms. Given the warning regarding singularity in the LMM with animal and cage as random factors, we opted to proceed with a simpler model that included only the animal as a random factor. Comparisons of model fits showed no significant difference in overall fit statistics between the models with and without the interaction between group and time, with the model without interaction having lower AIC as well as BIC values. The simpler model was chosen for analysis since it provided a better fit to the data.

**Ratio of the time spent on the open arms to the time spent on the closed arms:** A LMM (fixed factors: group, time; random factors: animal, cage) was used to analyze the ratio. Comparisons of model fits showed no significant difference in overall fit statistics between the models with and without the interaction between group and time, with the models without the interaction having lower AIC as well as BIC values. The simpler model was chosen for analysis since it provided a better fit to the data.

**Defecation:** A LMM (fixed factors: group, time; random factors: animal, cage) was used to analyze the distance travelled. Comparisons of model fits showed no significant difference in overall fit statistics between the models with and without the interaction between group and time, with the models without the interaction having lower AIC as well as BIC values. The simpler model was chosen for analysis since it provided a better fit to the data.

**Urination:** A LMM (fixed factors: group, time, interaction between group and time; random factors: animal, cage) was used to analyze the number of urinary spots counted after the Elevated Plus Maze Test. This decision was made after LMMs with and without interaction were fitted to the data and diagnostic plots were inspected. While the residuals of the LMM without interaction deviated from the normal distribution, the residuals of the LMM with interaction as fixed factor met the assumptions of the normal distribution. Therefore, the more complex model was chosen for analysis although the comparisons of model fits showed no significant difference in overall fit statistics between these two models. AIC values were lower in the model with interaction and BIC values lower in the model without interaction as fixed factor.

### Resting metabolic rate

For four animals of the LE group, there were not enough data points before the enrichment phase available for analysis. Therefore, these four mice were excluded. Values of the resting metabolic rate were logarithmized to meet the assumptions of a normal distribution and analyzed using A LMM (fixed factors: group, time; random factors: animal, cage). Comparisons of model fits showed no significant difference in overall fit statistics between the models with and without the interaction between group and time. AIC and BIC values were lower for the simpler model. The simpler model was chosen for analysis since it provided a better fit to the data.

### Performance in lockboxes in the calorimetric apparatus

**Number of mice solving the lockbox:** Due to warnings suggesting that the optimization algorithm encountered difficulties, we opted not to use a LMM or GLMM. Instead, the Chi-square test was used to compare the number of mice that were able to open the 2-step lockbox (binary parameter) between the three groups at each time point. Moreover, the number of mice that were able to solve the lockbox was compared between the last trial before and the trial after the enrichment phase for each group using the McNemar test.

**Total interaction time with the lockbox:** A LMM (fixed factors: group, time; random factors: animal, cage) was used to analyze the total interaction time with the lockbox. Comparisons of model fits showed no significant difference in overall fit statistics between the models with and without the interaction between group and time, with the models without the interaction having lower AIC as well as BIC values. The simpler model was chosen for analysis since it provided a better fit to the data.

**Interaction time with the lockbox until solved:** A LMM (fixed factors: group, time; random factor: animal) was employed to analyze the interaction time with the lockbox until solved among mice that were able to solve the lockbox. For this square root transformation of the values was conducted to meet the assumptions of a normal distribution. Given the warning regarding singularity in the LMM with animal and cage as random factors, we opted to proceed with a simpler model that included only the animal as a random factor. Comparisons of model fits showed no significant difference in overall fit statistics between the models with and without the interaction between group and time. AIC and BIC values were lower the simpler model. The simpler model was chosen for analysis since it provided a better fit to the data.

### Oxygen consumption during lockbox trial in the calorimetric apparatus

A LMM (fixed factors: group, time, interaction between group and time; random factors: animal, cage) was used to analyze the oxygen consumption during the lockbox trials in the calorimetric apparatus. Comparisons of model fits showed a significant difference in overall fit statistics between the models with and without the interaction between group and time. The model with the interaction between group and time had lower AIC, the model without the interaction had lower BIC values. We advocated for the use of the more complex model as it offered a more comprehensive and interpretable analysis of the data.

### Fecal corticosterone metabolites

A LMM (fixed factors: group, time; random factors: animal, cage) was used to analyze the FCM concentrations. Since the residuals of the model including the interaction between group and time were not normally distributed, the simpler model without the interaction as fixed factor was chosen for analysis.

### Adrenal glands

The percentage weight of the adrenal glands relative to the bodyweight measured on the last day of the experiments was calculated. A LMM (fixed factor: group; random factor: cage) was used for analysis.

### Bodyweight

A LMM (fixed factors: group, week of experiment; random factors: animal, cage) was used to analyze the bodyweight. Comparisons of model fits showed no significant difference in overall fit statistics between the models with and without the interaction between group and time. AIC values were lower for the complex model, while BIC values were lower for the simpler model. The simpler model was chosen for analysis since it provided a better fit to the data.

## Results

### Grid Exploratory Paradigm

The interrater reliability for the video analysis ranged from κ = 0.907 to κ = 0.966 which is considered almost perfect according to Landis and Koch ^59^.

**Total time of exploration:** In the LMM, the group had no significant effect (F _2, 11.09_ = 1.20, p = 0.338) on the total time of exploration. Time (F _1, 33_ = 19.73, p < 0.001) significantly affected the total time of exploration, with higher values before compared to after the enrichment phase. Moreover, the interaction between group and time (F _2, 33_ = 5.54, p = 0.008) had a significant effect on the total time of exploration.

**Figure 5.**
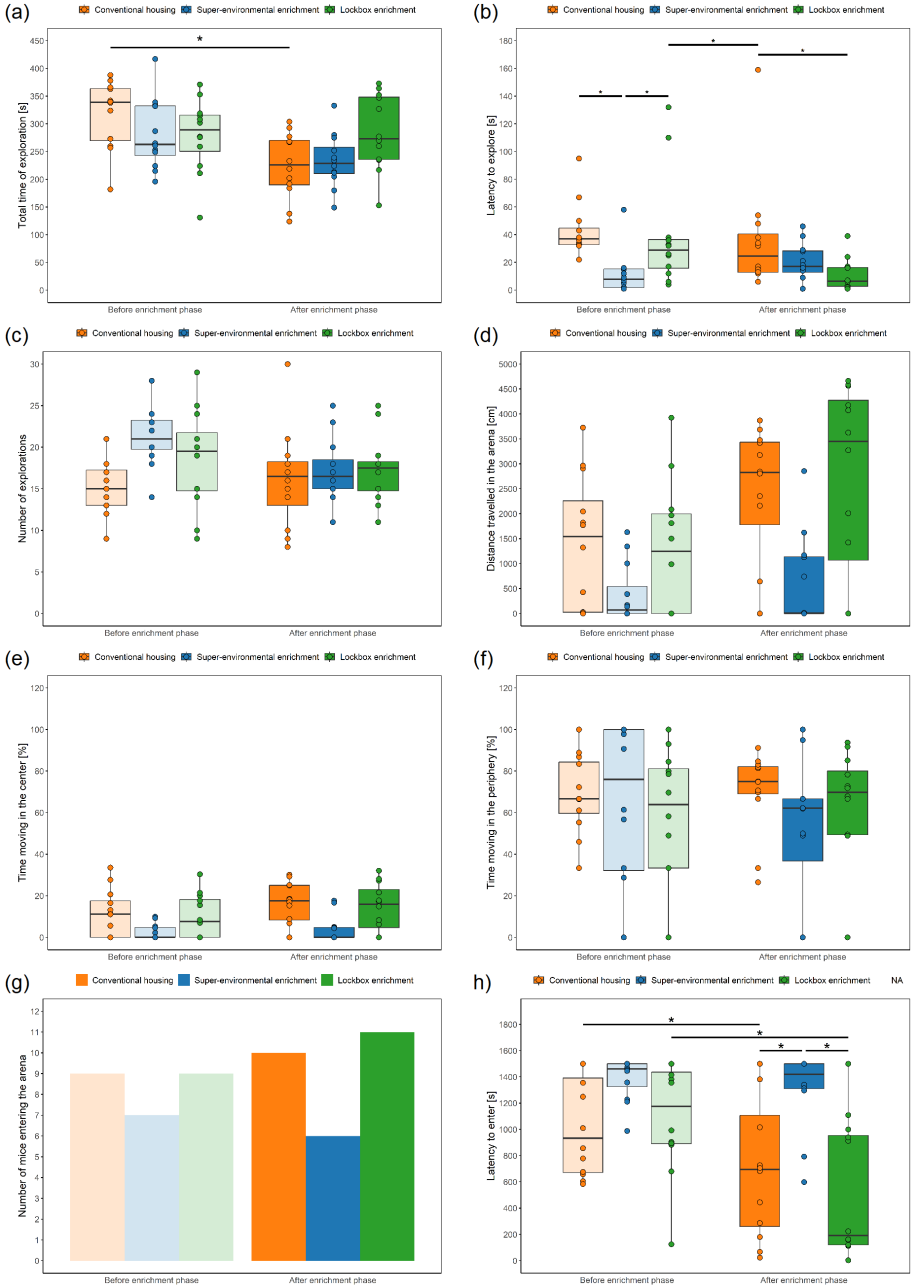
Grid Exploratory Paradigm und Free Exploratory Paradigm before and after exposure to the different types of enrichment. Boxplots: The box represents the interquartile range (IQR), box edges are the 25 ^th^ and 75 ^th^ quartile, and the whiskers represent values which are no greater than 1.5 × IQR. Additionally, individual data points representing each animal are overlaid on the boxplot as dots. It's important to note that these dots may overlap due to multiple animals having the same or very similar values. Grid Exploratory Paradigm: ( **a**) Total time of exploration, ( **b**) latency to explore, ( **c**) number of explorations; Free Exploratory Paradigm: ( **d**) distance travelled in the arena, ( **e**) percentage of time moving in the center, ( **f**) time moving in the periphery, ( **g**) number of mice entering the arena (i.e., a mouse entered the arena when all four paws left the tunnel and were placed on the floor of the arena on at least on day out of the three days on which the test was performed), ( **h**) latency to enter the arena (i.e., entering the arena was defined as placing all paws on the floor of the arena; if a mouse did not enter the arena, the latency was set to 1500 s). * p < 0.05 (linear mixed-effects model or generalized linear mixed-effects model, post-hoc pairwise comparisons of interaction between group and time), n = 12 animals per group. For ( **e**) and ( **f**) applied: if the DLCAnalyzer did not detect a mouse moving in the center or in the periphery on all three days, the mouse was excluded; n = 12 animals both before and after the enrichment phase in the CH group, n = 10 before and n = 9 animals after the enrichment phase in the SEE group, n = 10 animals both before and after the enrichment phase in the LE group were included in this analysis. This number may vary from the number of mice entering the arena with all four paws since we did not track the paws but only the body center using DLC.

Post-hoc analysis of the interaction effect revealed that the groups neither differed before (CH versus LE: estimate = 38.75, SE = 28.1, df = 11.1, t.ratio = 1.38, p = 0.326; CH versus SEE: estimate = 36.58, SE = 28.1, df = 11.1, t.ratio = 1.30, p = 0.330; LE versus SEE: estimate = –2.17, SE = 28.1, df = 11.1, t.ratio = –0.077, p = 0.940) nor after the enrichment phase (CH versus LE: estimate = –59.17, SE = 28.1, df = 11.1, t.ratio = –2.103, p = 0.205; CH versus SEE: estimate = –7.92, SE = 28.1, df = 11.1, t.ratio = –0.28, p = 0.919; LE versus SEE: estimate = 51.25, SE = 28.1, df = 11.1, t.ratio = 1.82, p = 0.205). The total time of exploration was higher before compared to after the enrichment phase in the CH group (estimate = 92.50, SE = 20.8, df = 33.0, t = 4.44, p = 0.001), while the values measured before and after the enrichment phase neither differed in the LE (estimate = –5.42, SE = 20.8, df = 33.0, t = –0.260, p = 0.919) nor the SEE group (estimate = 48.00, SE = 20.8, df = 33.0, t = 2.31, p = 0.138) ( Figure 5a).

**Latency to explore:** In the GLMM, the effects of group (Chi ^2^ = 15.57, df = 2, p < 0.001), and interaction between group and time (Chi ^2^ = 17.35, df = 2, p < 0.001) on the latency to explore were significant. Time had no significant effect (Chi ^2^ = 1.04, df = 1, p = 0.308).

Post-hoc analysis of the group revealed that CH differed compared to LE (estimate = 0.71, SE = 0.30, df = Inf, z.ratio = 2.34, p = 0.03) and SEE (estimate = 0.90, SE = 0.30, df = Inf, z.ratio = 3.03, p = 0.007). LE and SEE did not differ from each other (estimate = 0.20, SE = 0.30, df = Inf, z.ratio = 0.65, p = 0.52).

The post-hoc analysis of the interaction effect demonstrated that some group differences were based on differences between the groups at baseline and were not an effect of the different enrichment conditions. Before the enrichment phase, the latency to explore in mice assigned to SEE was lower than in mice assigned to CH (estimate = 1.41, SE = 0.39, df = Inf, z.ratio = 3.67, p = 0.001) or LE (estimate = 1.22, SE = 0.39, df = Inf, z.ratio = 3.16, p = 0.005) ( Figure 5b). There was no difference between CH and LE (estimate = = 0.20, SE = 0.38, df = Inf, z.ratio = 0.52, p = 0.696). After the enrichment phase, the latency to explore differed between CH and LE (estimate = 1.22, SE = 0.38, df = Inf, z.ratio = 3.18, p = 0.005), while there was no significant difference between CH and SEE (estimate = 0.40, SE = 0.38, df = Inf, z.ratio = 1.03, p = 0.385) or LE and SEE (estimate = –0.82, SE = 0.39, df = Inf, z.ratio = –2.09, p = 0.079). When comparing the latency to explore before and after the enrichment phase within the group, the values significantly decreased in the LE group (estimate = 1.35, SE = 0.33, df = Inf, z.ratio = 4.08, p < 0.001). There was no significant change over time within the CH group (estimate = 0.33, SE = 0.32, df = Inf, z.ratio = 1.02, p = 0.385) and SEE group (estimate = –0.68, SE = 0.35, df = Inf, z.ratio = –1.93, p = 0.089).

**Number of explorations:** In the LMM, neither group (F _2, 8.63_ = 3.32, p = 0.085) nor time (F _1, 33_ = 0.64, p = 0.428) significantly affected the number of explorations, while the interaction between group and time had a significant effect (F _2, 33_ = 3.365, p = 0.047). However, the post-hoc analysis of the interaction effect did not identify group differences before (CH versus LE: estimate = –3.67, SE = 2.50, df = 8.63, t.ratio = –1.47, p = 0.53; CH versus SEE: estimate = –6.42, SE = 2.50, df = 8.63, t.ratio = –2.57, p = 0.23; LE versus SEE: estimate = –2.75, SE = 2.50, df = 8.63, t.ratio = –1.10, p = 0.58) or after (CH versus LE: estimate = –1.17, SE = 2.50, df = 8.63, t.ratio = –0.47, p = 0.724; CH versus SEE: estimate = –1.08, SE = 2.50, df = 8.63, t.ratio = –0.43, p = 0.724; LE versus SEE: estimate = 0.08, SE = 2.50, df = 8.63, t.ratio = 0.03, p = 0.974) the enrichment phase were found ( Figure 5c). The comparison of the values measured before and after the enrichment phase within the groups did not reveal any significant differences (CH: estimate = –1.17, SE = 1.45, df = 33, t.ratio = –0.80, p = 0.584; SEE: estimate = 4.17, SE = 1.45, df = 33, t.ratio = 2.86, p = 0.108; LE: estimate = 1.33, SE = 1.45, df = 33, t.ratio = 0.92, p = 0.584).

### Free Exploratory Paradigm

The variances observed for the distance travelled were explained by the individual animal while group and batch did not significantly contribute to the observed variances ( Table 1). The repeatability value of 0.357 after the enrichment phase indicated that 35.7 % of the variance can be explained by the factor animal. Independent of the time point before or after the enrichment phase, the coefficient of variation determined for distance travelled was lowest on day 3 ( Table 2).

**Table 1.** Repeatability values for distance traveled in the Free Exploratory Paradigm, Open Field, and Elevated Plus Maze test with multiple grouping factors: animal, group, and batch. For the random factors animal, group, and batch, the repeatability R, the 95 % confidence interval (CI) with lower and upper bounds calculated at the 2.5 ^th^ and 97.5 ^th^ percentiles, and the p values calculated by the likelihood ratio test were determined over the three days (n = 36 mice). Distribution of Free Exploratory Paradigm data generated before the enrichment phase did not allow to analyze repeatability values. * Due to a technical malfunction of the recording from the lockbox enrichment group on day 1 after the enrichment phase, four mice of this group had to be excluded from the analysis.

Test	Repeatability for animal			Repeatability for group			Repeatability for batch		
	R	CI	p-value	R	CI	p-value	R	CI	p-value
Before enrichment phase									
Open Field Test	0.401	[0.125; 0.559]	< 0.001	0	[0; 0.142]	1	0	[0; 0.134]	1
Elevated Plus Maze Test	0.354	[0.12; 0.531]	< 0.001	0.056	[0; 0.25]	0.192	0	[0; 0.124]	1
After enrichment phase									
Free Exploratory Paradigm *	0.357	[0.144; 0.524]	< 0.001	0	[0; 0.076]	1	0.022	[0; 0.118]	0.316
Open Field Test	0.307	[0.066; 0.468]	0.001	0.021	[0; 0.158]	0.347	0	[0; 0.103]	0.5
Elevated Plus Maze Test	0.298	[0.059; 0.478]	0.001	0.017	[0; 0.172]	0.367	0.044	[0; 0.211]	0.221

**Table 2.** Coefficient of variation for the distance traveled in the Free Exploratory Paradigm, Open Field, and Elevated Plus Maze test. The data of 36 mice including all study groups are shown as the coefficient of variation, which was calculated using the following formula: (standard deviation/mean) × 100. The lowest value in a row was underlined. For the distance traveled, independent of the time point before or after the enrichment phase, the coefficient of variation was lowest on day 3 in the Free Exploratory Paradigm and the Open Field Test. In the Elevated Plus Maze Test, the coefficient of variation was lowest on day 2 before and on day 1 after the enrichment phase. *Due to a technical malfunction of the recording from the lockbox enrichment group on day 1 after the enrichment phase in the Free Exploratory Paradigm, four mice had to be excluded when calculating mean and standard deviation of the distance traveled on day 1.

Test	Day		
	1	2	3
Before enrichment phase			
Free Exploratory Paradigm	262	149	107
Open Field Test	17	16	13
Elevated Plus Maze Test	19	15	18
After enrichment phase			
Free Exploratory Paradigm *	170	111	79
Open Field Test	26	19	18
Elevated Plus Maze Test	23	27	26

**Figure 6.**
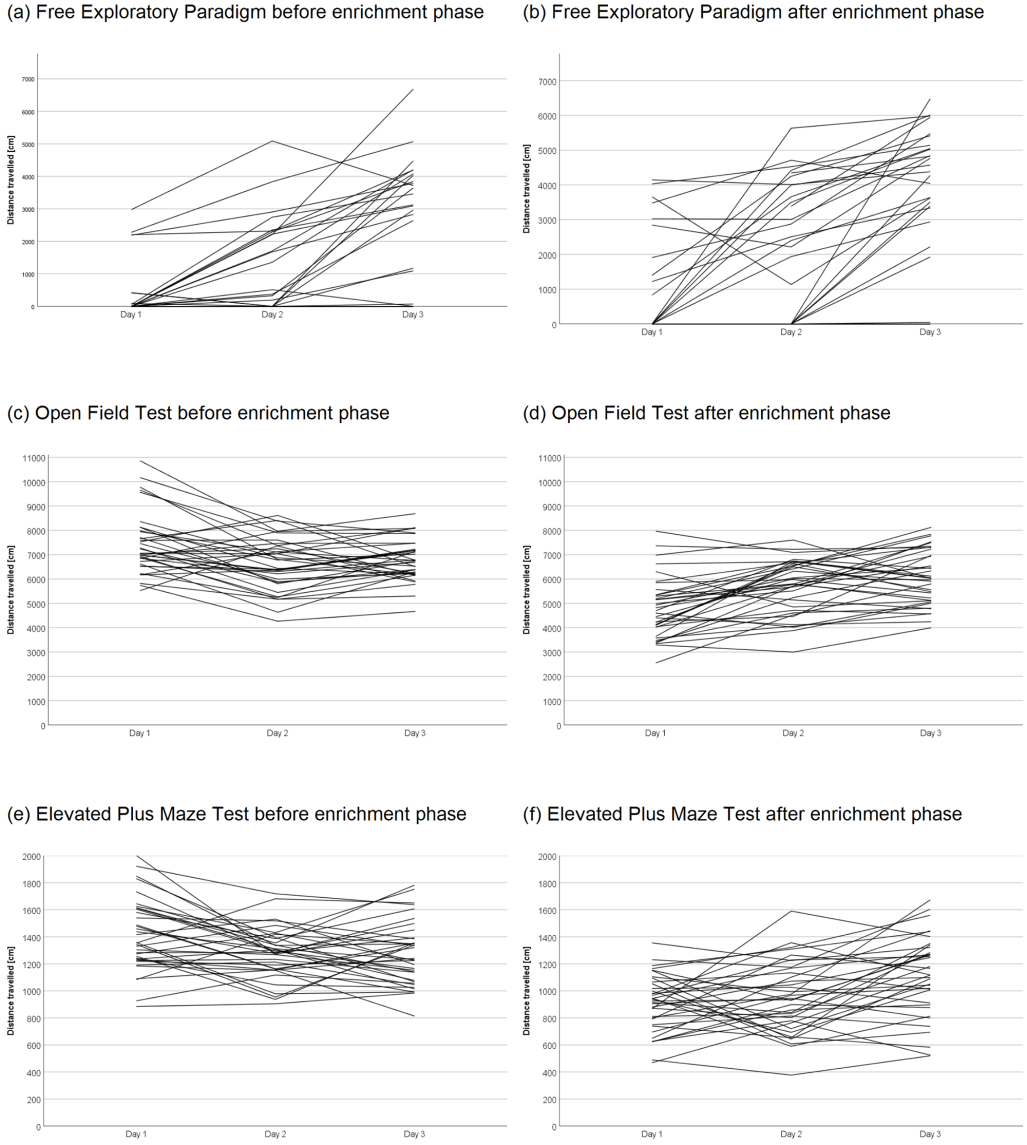
Distance traveled in the Free Exploratory Paradigm, Open Field Test, and Elevated Plus Maze Test during habituation. Data are presented as exact values for each mouse. Free Exploratory Paradigm: ( **a**) Distance traveled in the Free Exploratory Paradigm decreased before (n = 36 animals) and ( **b**) after (n = 36 animals) the enrichment phase during habituation. ( **c**) Distance traveled in the Open field test decreased before (n = 36 animals) the enrichment phase and ( **d**) increased after (n = 36) enrichment phase during habituation. ( **e**) Distance traveled in the Elevated Plus Maze Test did not change over time before but ( **f**) increased after the enrichment phase during habituation. Data were analyzed using a Friedman test with Dunn-Bonferroni post-hoc test. Significant differences between the days are described in the results section.

Figure 6a shows that before the mice were exposed to the enrichment, the distance traveled increased over time (Friedman test: Chi ^2^ = 25.91, df = 2, p < 0.001, n = 36), with the mice moving more on day 3 when compared to day 1 (p < 0.001, r = 0.15). After the exposure to the enrichment, four mice from the lockbox enrichment group had to be excluded from this analysis because of technical malfunctions of the camera on day 1. The distance traveled increased over the three days after the enrichment phase (Chi ^2^ = 32.64, df = 2, p < 0.001, n = 32) ( Figure 6b). The animals moved more on day 3 when compared to day 1 (p < 0.001, r = 0,18) and day 2 (p = 0.002, r = 0.14).

For the following analyses, the data generated on day 1, day 2, and day 3 were averaged.

**Distance travelled in the arena:** In the LMM, neither group (F _2, 54.67_) = 2.15, p = 0.126) nor the interaction between group and time (F _2, 33_ = 2.30, p = 0.117) had a significant effect on the distance travelled in the arena ( Figure 5d). However, time was found to have a significant effect on the distance travelled (F _2, 33_ = 5.75, p = 0.022), with higher values after than before the enrichment phase.

**Percentage of time moving in the center:** While the LMM did not reveal group differences in the percentage of time moving in the center (F _2, 6_ = 4.77, p = 0.058), time had a significant effect (F _1, 35_ = 4.95, p = 0.033). The mice moved more in the center after than before the enrichment phase ( Figure 5e).

**Percentage of time moving in the periphery:** Neither group (F _2, 6_ = 0.55, p = 0.602) nor time (F _1, 35_ = 0.31, p = 0.582) significantly affected the percentage of time moving in the periphery in the LMM ( Figure 5f).

**Number of mice entering the arena:** In the GLMM, neither group (Chi ^2^ = 2.83, df = 2, p = 0.243) nor time (Chi ^2^ = 0.79, df = 1, p = 0.374) significantly affected whether a mouse entered the arena with all four paws on at least one day out of the three days the test was performed ( Figure 5g).

**Latency to enter the arena:** In the LMM, the latency to enter the arena with all four paws was not affected by the group (F _2, 51.07_ = 2.6918, p = 0.077). Time (F _1, 33_ = 7.68, p = 0.009) and the interaction between group and time (F _2, 33_ = 4.542, p = 0.018) had significant effects on the latency. The latency to enter the arena was higher before compared to after the enrichment phase.

Post-hoc analysis of the interaction effect showed that, before the enrichment phase, the groups did not differ (CH versus LE estimate: –71.7, SE = 167, df = 9.95, t.ratio = –0.43, p = 0.676; CH versus SEE: estimate = –365.0, SE = 167, df = 9.95, t.ratio = –2.19, p = 0.089; LE versus SEE: estimate = –293.3, SE = 167, df = 9.95, t.ratio = –1.760, p = 0.164) ( Figure 5h). After the enrichment phase the mice housed under SEE took longer to enter the open arena than mice with access to LE (estimate = –774.8, SE = 167, df = 9.95, t.ratio = –4.65, p = 0.005) and CH mice (estimate = –595.1, SE = 167, df = 9.95, t.ratio = –3.57, p = 0.015). There was no difference between CH and LE (estimate = 179.7, SE = 167, df= 9.95, t.ratio = 1.08, p = 0.354). When comparing the latency to enter the arena within the groups over time, a significant decrease was found in CH (estimate = 313.1, SE = 113, df = 33, t.ratio = 2.77, p = 0.023) and LE (estimate = 564.4, SE = 113, df = 33, p.ratio = 5.00, p < 0.001) but not SEE (estimate = 83.0, SE = 113, df = 33, t.ratio = 0.74, p = 0.50).

**Urination and defecation:** All mice except from one mouse, that was housed under conventional conditions and excreted one fecal bolus on day 3 after the enrichment phase, neither defecated nor urinated in the arena.

### Open Field Test

The variances observed for the distance travelled were explained by the individual animal while group and batch did not significantly contribute to the observed variances ( Table 1). The repeatability values of 0.401 (before enrichment phase) and 0.307 (after enrichment phase) indicated that 40.1 % and 30.7 % of the variance can be explained by the factor animal. Independent of the time point before or after the enrichment phase, the coefficient of variation determined for distance travelled was lowest on day 3 ( Table 2).

Figure 6c illustrates that before the enrichment phase, the distance traveled differed between the three days (Friedman test: Chi ^2^ = 11.72, df = 2, p = 0.003, n = 36), with higher values on day 1 when compared to day 2 (p = 0.002, r = 0.13). After the mice were exposed to the enrichment ( Figure 6d), the distance traveled (Chi ^2^ = 17.17, df = 2, p < 0.001, n = 36) differed between the three days. The mice moved more on day 2 (p = 0.004, r = 0.13) as well as day 3 (p < 0.001, r = 0.15) in comparison to day 1.

For the following analysis, data generated on day 1, day 2, and day 3 were averaged.

**Figure 7.**
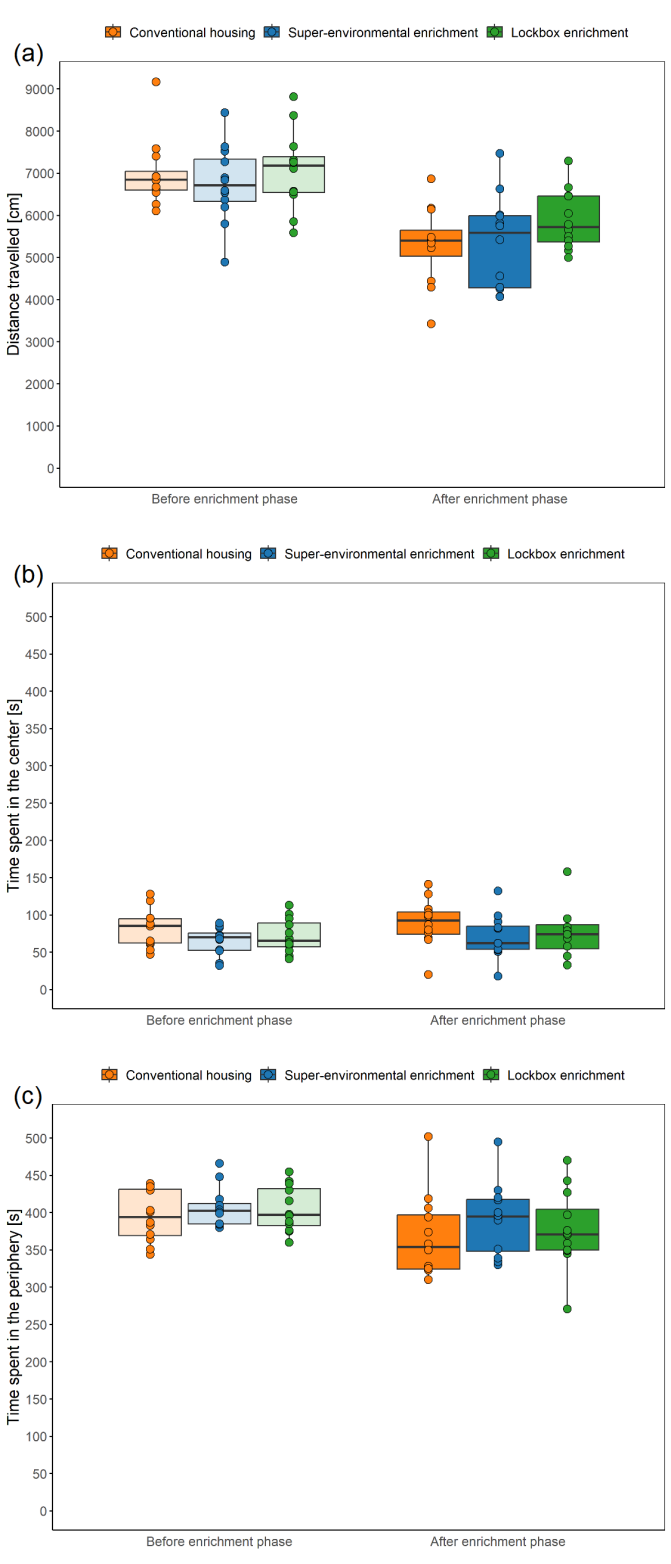
Open Field Test before and after the enrichment phase. Boxplots: The box represents the interquartile range (IQR), box edges are the 25 ^th^ and 75 ^th^ quartile, and the whiskers represent values which are no greater than 1.5 × IQR. Additionally, individual data points representing each animal are overlaid on the boxplot as dots. It's important to note that these dots may overlap due to multiple animals having the same or very similar values. ( **a**) Distance travelled, ( **b**) time spent in the center, ( **c**) time spent in the periphery; n = 12 animals per group.

**Distance travelled:** The LMM revealed that time had a significant effect on the distance travelled (F _1, 35_ = 89.53, p < 0.001), with higher values before when compared to after the enrichment phase ( Figure 7a). Group (F _2, 33_ = 0.96, p = 0.392) did not significantly influence the distance travelled.

**Time spent in the center:** In the LMM, neither group (F _2, 6_ = 1.42, p = 0.312) nor time (F _1, 35_ = 1.03, p = 0.318) significantly affected the time spent in the center ( Figure 7b).

**Time spent in the periphery:** The time spent in the periphery was not affected by group (F _2, 11.21_ = 0.87, p = 0.445), time (F _1, 33_ = 0.61, 0.441), or the interaction between group and time (F _2, 33_ = 0.04, p = 0.965) in the LMM ( Figure 7c).

**Urination:** Given as median and 25 ^th^–75 ^th^ percentiles in brackets, 0 (0–0) urine spots before and 0 (0–0) urine spots after the enrichment phase in mice assigned to CH, 1 (0–1) urine spots before and 0 (0–1) urine spots after the enrichment phase in mice assigned to SEE, as well as 1 (0–1) urine spots before and 0 (0–1) urine spots after the enrichment phase in mice assigned to LE were counted after the Open Field Test (the values were rounded up to whole number). The GLMM revealed that neither group (Chi ^2^ = 5.64, df = 2, p = 0.060) nor time (Chi ^2^ = 0.07, df = 1, p = 0.797) significantly affected urination in the Open Field Test.

**Defecation:** Given as median and 25 ^th^–75 ^th^ percentiles in brackets, mice assigned to CH excreted 1 (1–2) fecal boli before and 2 (2–2) fecal boli after the enrichment phase, mice assigned to SEE excreted 3 (2– 4) fecal boli before and 3 (2–4) fecal boli after the enrichment phase, and mice assigned to LE excreted 3 (1–5) fecal boli before and 3 (2–4) fecal boli after the enrichment phase during the Open Field test (the values were rounded up to whole number). The groups (Chi ^2^ = 6.97, df = 2, p = 0.031) significantly differed in the GLMM, while time (Chi ^2^ = 0.02 , df = 1, p = 0.882) had no significant effect on the number of fecal boli excreted during the Open Field Test. In the CH group significantly fewer fecal boli were excreted compared to the LE group (estimate = –0.47, SE = 0.20, df = Inf, z.ratio = –2.41, p = 0.030) and the SEE group (estimate –0.46, SE = 0.20, df = Inf, z.ratio = –2.33, p = 0.0298). When inspecting the data, it can be noticed that mice assigned to CH excreted fewer pellets both before and after the enrichment phase, suggesting that this observation was not attributable to the type of enrichment.

### Elevated Plus Maze Test

The variances observed for the distance travelled were explained by the individual animal while group and batch did not significantly contribute to the observed variances ( Table 1). The repeatability values of 0.354 (before enrichment phase) and 0.298 (after enrichment phase) indicated that 35.4 % and 29.8 % of the variance can be explained by the factor animal. The coefficient of variation calculated for distance travelled was lowest on day 2 before and on day 1 after the enrichment phase ( Table 2).

Figure 6e demonstrates that the distance traveled did not differ between the three days before exposure to the enrichment (Friedman test: Chi ^2^ = 4.22, df = 2, p = 0.121, n = 36). After the enrichment phase ( Figure 6f), the distance traveled significantly differed between the three days (Chi ^2^ = 8.67, df = 2, p = 0.013, n = 36). The mice moved a higher distance on day 3 when compared to day 1 (p = 0.014, r = 0.11).

For the following analysis, the mean of the three days was calculated.

**Figure 8.**
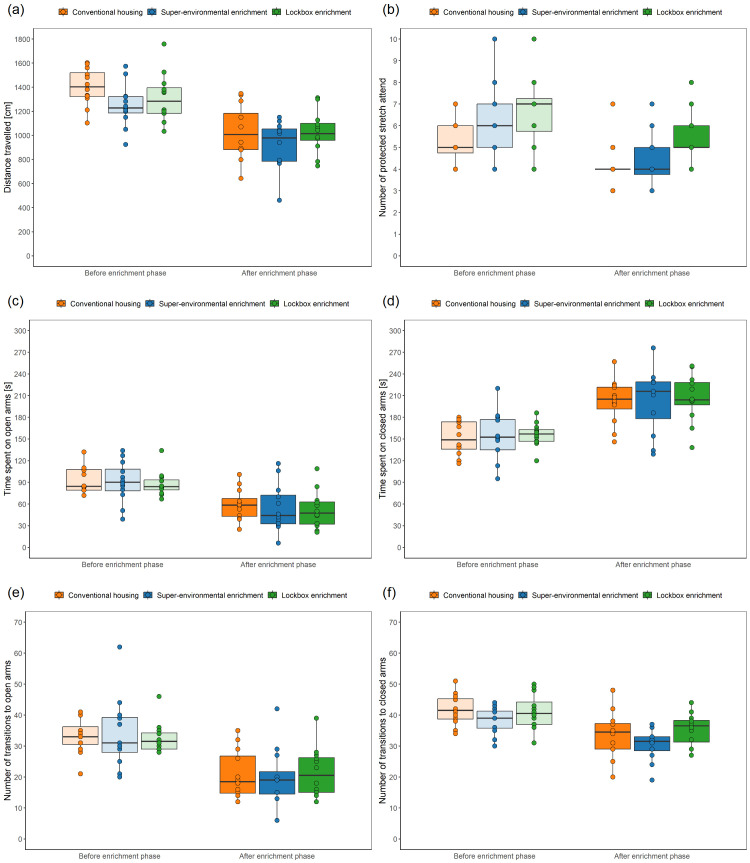
Elevated Plus Maze Test before and after the enrichment phase. Boxplots: The box represents the interquartile range (IQR), box edges are the 25 ^th^ and 75 ^th^ quartile, and the whiskers represent values which are no greater than 1.5 × IQR. Additionally, individual data points representing each animal are overlaid on the boxplot as dots. It's important to note that these dots may overlap due to multiple animals having the same or very similar values. ( **a**) Distance travelled in the arena, ( **b**) number of protected stretch attend, ( **c**) time spent on open arms, ( **d**) time spent on closed arms, ( **e**) number of transitions to open arms, ( **f**) number of transitions to closed arms; n = 12 animals per group.

**Distance travelled:** In the LMM, the distance travelled was significantly affected by the time (F _1, 35_ = 125.87, p < 0.001), with higher values before when compared after the enrichment phase ( Figure 8a). Group (F _2, 6_ = 1.116, p = 0.387) had no significant effect.

**Number of protected stretch attend:** The number of protected stretch attend was significantly affected by group (F _2, 33_ = 7.41, p = 0.002) and time (F _1, 35_ = 19.83, p < 0.001) in the LMM ( Figure 8b). The number of protected stretch attend was significantly higher before compared to after the enrichment phase. However, post-hoc pairwise comparisons did not reveal any significant group differences (LE versus CH: estimate = –1.54, SE = 0.51, df = 6, t.ratio = -3.02, p = 0.070; CH versus SEE: estimate: –0.63, SE = 0.51, df = 6, t.ratio = –1.23, p = 0.267; LE versus SEE: estimate = 0.92, SE = 0.51, df = 6, t.ratio = 1.80, p = 0.184).

**Time spent on open arms:** While group (F _2, 6_ = 0.18, p = 0.842) had no effect, time (F _1, 35_ = 149.20, p < 0.001) significantly decreased the time spent on the open arms in the LMM ( Figure 8c). Values were higher before than after the enrichment phase.

**Time spent on closed arms:** While the time spent on the closed arms was not affected by group (F _2, 6_ = 0.04, p = 0.957), the time had a significantly increasing effect (F _1, 35_ = 147.18, p < 0.001) in the LMM, with higher values after than before the enrichment phase ( Figure 8d).

**Number of transitions to open arms:** The GLMM showed that the number of transitions to the open arms was not significantly influenced by group (Chi ^2^ = 0.05, df = 2, p = 0.975) but by time (Chi ^2^ = 104.18, df = 1, p < 0.001) ( Figure 8e). Values were higher before than after the enrichment phase.

**Number of transitions to closed arms:** Group did not influence the number of the transitions to the closed arms (F _2, 33_ = 3.14, p = 0.057), while time had a significantly decreasing effect (F _1, 35_ = 39.44, p < 0.001) in the LMM ( Figure 8f). The values were higher before than after the enrichment phase.

**Ratio of the time spent on the opens arms to the time spent on the closed arms:** Given in median and 25 ^th^–75 ^th^ percentiles in brackets, before the exposure to the different types of enrichment, the ratio was 0.55 (0.47–0.83), 0.59 (0.42–0.84), and 0.53 (0.47–0.65) in mice assigned to CH, SEE, and LE, respectively. After the enrichment phase, the ratio was 0.28 (0.18–0.42) in the CH group, 0.20 (0.14–0.48) in the SEE group, and 0.23 (0.14–0.33) in the LE group. Group (F _2, 6_ = 0.17, p = 0.848) had no effect, but time (F1, 35 = 130.33, p < 0.001) significantly decreased the ratio in the LMM, with higher values before compared to after the enrichment phase.

**Defecation:** Given as median and 25 ^th^–75 ^th^ percentiles in brackets, mice assigned to CH excreted 1 (0–1) fecal boli before and 2 (2–3) fecal boli after the enrichment phase, mice assigned to SEE excreted 2 (1–3) fecal boli before and 2 (1–3) fecal boli after the enrichment phase, mice assigned to LE excreted 1 (0–2) fecal boli before and 3 (3–4) fecal boli after the enrichment phase during the Elevated Plus Maze Test (the values are rounded up to whole number). While the number of fecal boli was not affected by group (F _2, 6_ = 0.18, p = 0.843), it significantly increased over time (F _1, 35_ = 25.97, p < 0.001). The animals excreted more feces during the Elevated Plus Maze Test after compared to before the enrichment phase.

**Urination:** Given as median and 25 ^th^–75 ^th^ percentiles in brackets, 0 (0–1) urine spots before and 0 (0–1) urine spots after the enrichment phase in mice assigned to CH, 1 (0–1) urine spots before and 0 (0–1) urine spots after the enrichment phase in mice assigned to SEE, 1 (0–1) urine spots before and 1 (1–1) urine spots after the enrichment phase in mice assigned to LE were counted after the Elevated Plus Maze Test (the values are rounded up to whole number). Neither group (F _2, 11.13_ = 0.40, p = 0.680), time (F _1, 33_ = 0.00, p = 1.000) nor interaction between group and time (F _2, 33_ = 2.70, p = 0.08) significantly affected the number of urinary spots counted after the Elevated Plus Maze Test.

### Resting metabolic rate

**Figure 9.**
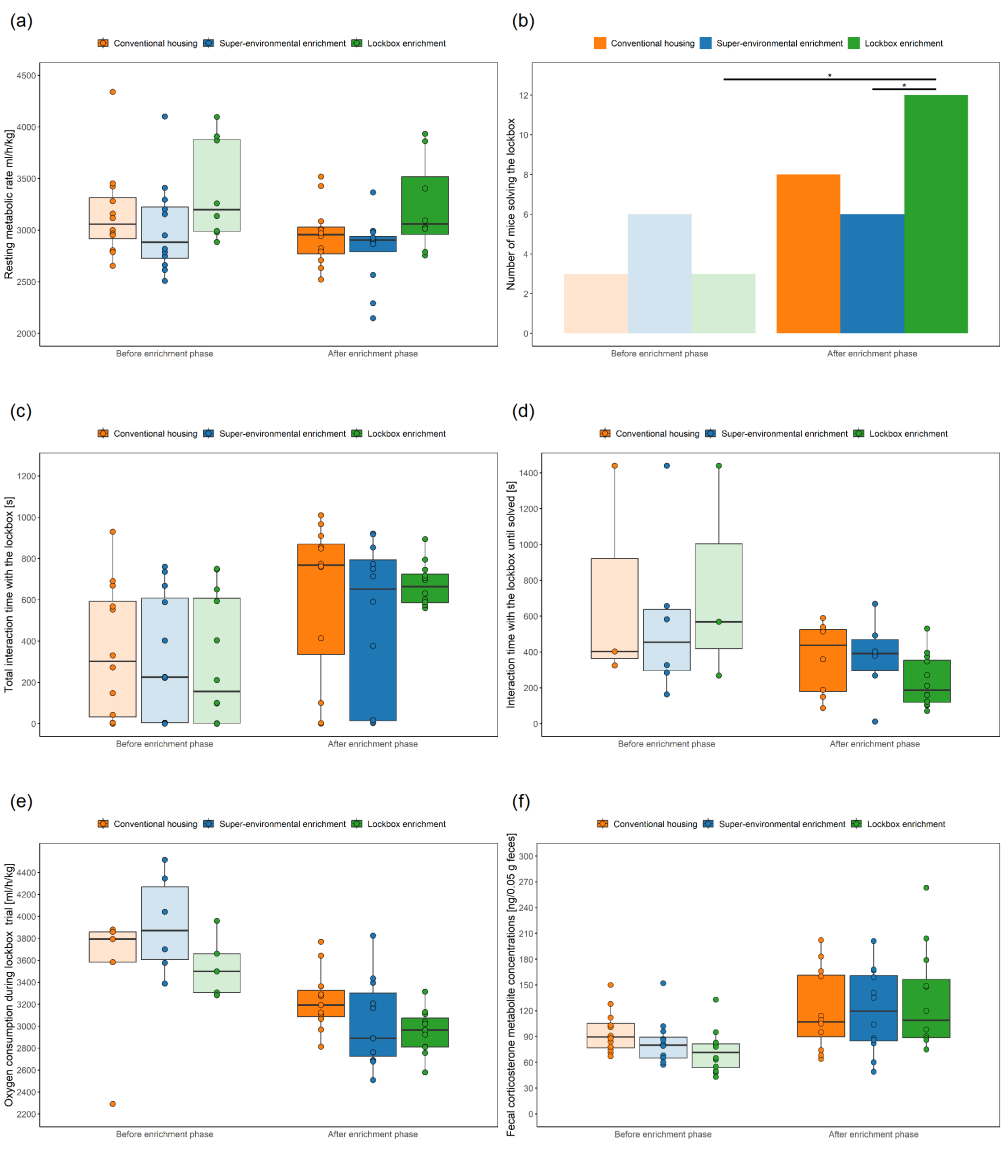
Calorimetric measurement before and after the enrichment phase. Boxplots: The box represents the interquartile range (IQR), box edges are the 25 ^th^ and 75 ^th^ quartile, and the whiskers represent values which are no greater than 1.5 × IQR. Additionally, individual data points representing each animal are overlaid on the boxplot as dots. It's important to note that these dots may overlap due to multiple animals having the same or very similar values. ( **a**) Resting metabolic rate (convention housing: n = 12 animals, super-environmental enrichment: n = 12 animals, lockbox enrichment: n = 8 animals; 4 animals of the LE group were excluded because not enough data points of the measurement before the enrichment phase were available for analysis), ( **b**) number of mice solving the lockbox: n = 12 animals per group (data were analyzed using Chi-square test or McNemar test: * p < 0.05), ( **c**) total interaction time with the lockbox (n = 12 animals per group), ( **d**) interaction time with the lockbox until solved (only mice that were able to solve the lockbox were included; for n number see ( **b**)); ( **e**) oxygen consumption during lockbox trials in the calorimetric apparatus (only mice that interacted with the lockboxes and did not rest were included; before enrichment phase: n = 5 animals in the CH group, n = 6 animals in the SEE group, and n = 5 animals in the LE group; after the enrichment phase: n = 11 animals per group), ( **f**) fecal corticosterone metabolite concentrations (n = 12 animals per group).

In the LMM, time (F _1, 31_ = 4.49, p = 0.042) significantly affected the RMR, while group had no significant effect (F _2, 5_ = 3.88, p = 0.096) ( Figure 9a). The RMR was higher before compared to after the enrichment phase.

### Performance in lockbox trials in the calorimetric apparatus

The interrater reliability for the video analysis ranged from κ = 0.648 to κ = 0.885 which is considered substantial to almost perfect according to Landis and Koch ^59^.

**Number of mice solving the lockbox:** Before the exposure to the enrichment, the performance in opening the 2-step lockbox did not differ between the groups (Chi ^2^ = 2.250, df = 2, p = 0.490, Cramer-V = 0.250) ( Figure 9b). After the exposure to the enrichment, the three different groups differed from each other (Chi ^2^ = 7.754, df = 2, p = 0.031, Cramer-V = 0.464): All mice (n = 12) from the LE, 6 mice from the SEE and 8 mice from the CH group succeeded in solving this lockbox. When comparing the number of mice that were able to solve the lockboxes between the lockbox trial before and after the enrichment phase within the groups, the McNemar test revealed that only LE had a significant effect (Chi ^2^ = 7.11, df = 1, p = 0.004, n = 12; CH: Chi ^2^ = 3.20, df = 1, p = 0.063, n = 12; SEE: Chi ^2^ = 0, df = 1, p = 1.000, n = 12).

**Total interaction time with the lockbox:** While the group did not affect the interaction time with the lockboxes (F _2, 6_ = 0.17, p = 0.844), time had a significant effect (F _1, 35_ = 22.58, p < 0.001) in the LMM ( Figure 9c). The mice interacted for a longer time duration with the lockbox after compared to before the enrichment phase.

**Interaction time with the lockbox until solved:** Among the mice that were able to solve the lockbox, time significantly affected the interaction time with the lockbox until it was solved (F _1, 19.35_ = 12.67, p = 0.002) in the LMM ( Figure 9d). Group had no significant effect (F _2, 26.38_ = 0.53, p = 0.594). The interaction time with the lockbox until solved was decreased after compared to before the enrichment phase.

### Oxygen consumption during lockbox trial in the calorimetric apparatus

In the LMM, neither group (F _2, 16.59_ = 1.14, p = 0.344), time (F _1, 27.38_ = 2.13, p = 0.156) nor the interaction between group and time (F _2, 27.74_ = 2.84, p = 0.075) significantly affected the oxygen consumption during the lockbox trials ( Figure 9e).

### Fecal corticosterone metabolites

The LMM revealed that group (F _2, 6_ = 0.12, p = 0.892) had no significant effect on the FCM concentrations, but the time (F1, 35 =32.89, p < 0.001) significantly affected the values ( Figure 9f). The FCM values were higher after the exposure to the different types of enrichment compared to baseline.

### Bodyweight

**Figure 10.**
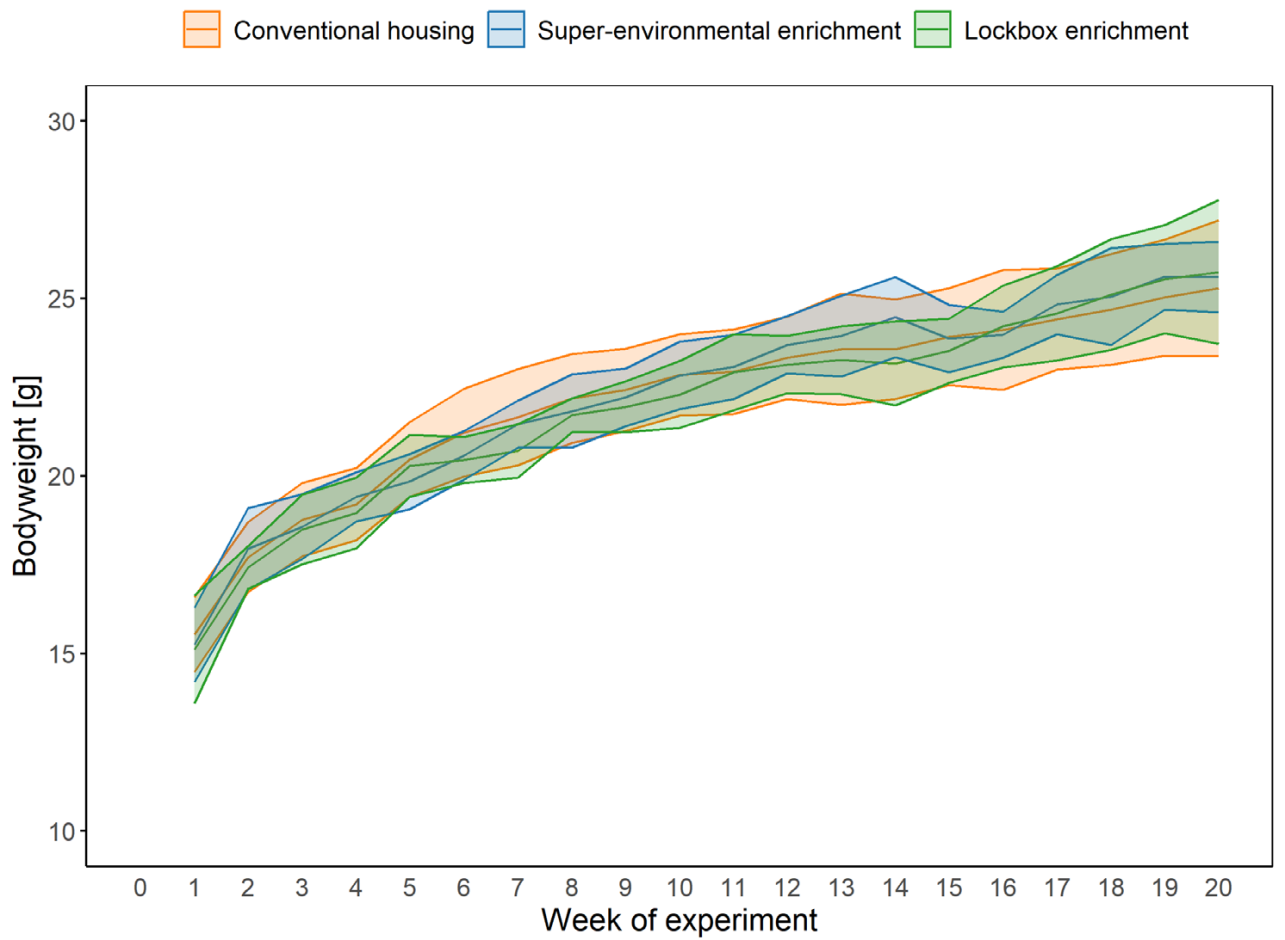
Bodyweight throughout the entire experiment. Data are given as mean ± standard deviation.

While group (F _2, 6_ = 0.30, p = 0.748) did not significantly affect the bodyweight in the LMM, week of experiment (F _1, 683_) = 3766.77, p < 0.001) had a significant effect, with increasing weight over time ( Figure 10).

### Adrenal glands

The percentage weight of the adrenal glands relative to the bodyweight measured in week 20 was not affected by the group in the LMM (F _2, 6_ = 0.16, p = 0.858; Table 3).

**Table 3.** Percentage weight of the adrenal glands relative to the bodyweight measured in week 20. Data are given as median and 25 ^th^–75 ^th^ percentiles in brackets; the values are rounded up; n = 12 mice per group.

Parameter	Conventional housing	Super-environmental enrichment	Lockbox enrichment
Weight of adrenal glands relative to bodyweight [%]	0.022 (0.021–0.024)	0.021 (0.020–0.026)	0.022 (0.018–0.025)

### Pathohistological evaluation

The toxicopathological analysis yielded no evidence of group-specific pathological alterations in the analyzed tissues. A mild, multifocal, chronic, interstitial, lymphocytic nephritis was noticed in some animals of all groups which was interpreted as a typical background pathology. 

### Coefficient of variation

The coefficient of variation for 23 continuous variables is shown in Table 4. For most variables, the coefficient of variation increased. However, the coefficient of variation decreased after the enrichment phase when compared to the time before the enrichment phase in 6 (CH), 7 (SEE), or 9 (LE) variables, indicating only slight differences between the study groups.

**Table 4.** Coefficient of variation for continuous variables examined before and after the enrichment phase. The data of each study group (n = 12 mice per group) are shown as the coefficient of variation, which was calculated using the following formula: (standard deviation/mean) × 100. If the coefficient of variation decreased within a group when compared to baseline (i.e., before the enrichment phase), the value was underlined. Values were rounded up to two decimal places. * The mean of the three testing days was used.

Test	Variable	Conventional housing		Super-environmental enrichment		Lockbox enrichment	
		Before enrichment phase	After enrichment phase	Before enrichment phase	After enrichment phase	Before enrichment phase	After enrichment phase
Grid Exploratory Paradigm	Total duration of exploration [s]	19,42	26,22	22,67	20,80	23,75	24,14
	Latency to explore [s]	48,16	115,66	135,41	72,09	102,22	107,69
	Number of explorations	21,22	36,74	18,56	22,33	31,68	23,42
Free Exploratory Paradigm *	Distance traveled	93,78	59,19	151,74	146,13	104,98	70,73
	Latency to enter the arena	36,74	76,13	11,80	23,00	38,86	98,98
	Time moving in the center [%]	97,77	64,61	144,83	160,40	106,18	79,75
	Time moving in the periphery [%]	27,54	28,59	62,22	67,05	60,09	52,11
Open Field Test	Distance travelled [cm]	17,31	17,31	13,70	20,69	13,29	11,90
	Time spent in the center [s]	30,85	35,03	28,47	41,78	31,92	43,00
	Time spent in the periphery [s]	8,64	15,46	6,58	12,24	7,58	13,85
Elevated Plus Maze Test	Distance travelled [cm]	11,07	21,95	14,21	21,27	15,31	16,85
	Time spent in the center [s]	17,70	28,15	17,78	35,97	13,16	27,81
	Number of transitions to the center	8,95	25,65	16,05	24,50	12,00	18,96
	Time spent on the open arms [s]	21,87	36,78	31,12	59,84	19,75	49,47
	Number of transitions to the open arms	17,58	36,86	33,63	47,04	15,10	36,94
	Time spent on the closed arms [s]	15,23	15,38	21,73	21,78	10,63	16,10
	Number of transitions to the closed arms	33,29	22,50	11,27	16,56	13,77	14,58
	Ratio of the time spent on the opens arms to the time spent on the closed arms	37,88	53,13	53,03	87,50	32,76	71,43
	Number of protected stretch attend	20,00	22,38	33,33	30,73	28,57	22,70
Resting metabolic rate	Oxygen consumption [ml/h/kg]	14,10	10,00	14,73	11,69	14,37	11,99
Performance in opening a 2-step lockbox	Total interaction time with the lockbox [s]	142,83	57,92	94,59	51,71	65,54	53,80
Oxygen consumption during the first 30-min lockbox trial in the calorimetric apparatus	Oxygen consumption [ml/h/kg]	13,85	8,46	11,88	10,88	8,29	8,24
Analysis of stress hormone metabolite concentrations	Fecal corticosterone metabolites concentrations [ng/0.05g feces]	25,93	38,33	31,17	40,38	34,32	43,95

## Discussion

In the scope of animal welfare, we investigated whether LE allowing both manipulative and cognitive activities affected the phenotype and influenced the affective state of young adult female laboratory mice when compared to animals kept under conventional or super-environmentally enriched housing conditions. Main findings of our study were that 2-month exposure to the different types of enrichment affected trait anxiety-related behavior and the performance in solving novel lockbox tasks. No effects were found in standard behavioral tests for state anxiety-related behavior and physiological variables, such as bodyweight, resting metabolic rate, stress hormone metabolite concentrations, and adrenal gland weights. The relative variability of most variables increased over time, independent of the housing conditions, while it decreased for some variables, particularly in animals with LE.

When interpreting the data of the three study groups, i.e., LE, SEE, and CH, it must be considered that in our study the conventional housing cage may provide the animals with more resources than what is usual in other facilities. All animals, including the CH group, were provided with more space, than required by law ^3^. In addition to nesting material, the “conventional housing” cages were equipped with a shelter and a handling tunnel. This may not accurately reflect the conditions of “conventional housing” in all laboratory animal facilities, which are often more impoverished, but was necessary for the purpose of our study. Otherwise, the differentiation between effects attributed to space and those attributed to enrichment would have been unfeasible.

### Neither lockbox nor super-environmental enrichment affected state anxiety-related behavior and physiological variables

The influence of enrichment on the phenotype of laboratory animals is subject to critical discussion because researchers are concerned that even minor changes to the cage environment may impact the data they are generating ^63^. In light of this, the absence of differences between our study groups in the Open Field and the Elevated Plus Maze Test, bodyweight, resting metabolic rate, stress hormone metabolite concentrations, and adrenal gland weights may alleviate these concerns. We could only detect two minor differences between the groups (i.e., defecation in the Open Field Test and number of protected stretch attend in the Elevated Plus Maz Test) that are negligible. Differences in defecation appeared to be attributed to baseline variations among the groups and the analysis of the number of protected stretch attend revealed group differences, but post-hoc pairwise comparisons did not identify any significant differences between groups.

Our findings are consistent with Bailoo *et al.* who also did not observe any effect of environmental enrichment on the time spent on the open arms of the Elevated Plus Maze or the time spent in the center of the Open Field in female C57BL/6JRj mice ^6^. Additionally, this research group did not find differences in the FCM concentrations or adrenal gland weights between female C57BL/6JRj mice housed under various types of enrichment, ranging from barren to SEE ^6^. In our study, all cages, regardless of the group, were equipped with transparent handling tunnels, which can also be considered as an enrichment item. Previous research has demonstrated that the provision of a transparent handling tunnel in a cage does not affect the Open Field Test, Elevated Plus Maze Test, plasma corticosterone concentrations, or adrenal gland weights in female C57BL/6J mice ^64^. However, the impact of enrichment on anxiety-related behavior and physiological stress variables of mice may depend on factors such as strain, sex, or age. Contradictory results were obtained in male C3H⁄eB mice, with environmental enrichment increasing the time spent in the center of the Open Field and elevating corticosterone concentrations ^65^. Moreover, environmental enrichment elevated the time spent in the center of the Open Field in male HAB mice ^66^ and the time spent in the open arms of the Elevated Plus Maze in wild type littermates of 5-HT _1A_R KO mice on a C57BL/6 background (sex unspecified) ^67^. In another study with a relatively small sample size, female C57BL/6J mice appeared to spend more time in the center of the Open Field when they were housed in larger enriched (n = 7) compared to smaller impoverished cages (n = 10) ^68^. Dickson *et al.* reported that female C57BL/6J mice housed in groups in a larger, enriched cage spent more time in the closed quadrants in the Elevated Zero Mazes compared to animals individually housed in a small, impoverished cage ^69^. In the latter study, it can be assumed that space and social structure played critical roles in addition to physical/inanimate enrichment items. In contrast, in our study we ensured that space and social structure were consistent across all study groups.

The method of FCM analysis used in our study has previously revealed a correlation between elevated FCM levels and a range of stressors ^70– 74^. The zenith of FCM concentrations typically occurs 8–10 hours post-exposure to a stressor, contingent upon the intestinal transit time ^49^. To mitigate the potential impact of the circadian rhythm on FCM excretion ^48^, all fecal samples excreted over a 24-hour period were collected. The FCMs concentrations measured after the 2-month enrichment phase suggested that the type of enrichment did not significantly affect the hypothalamic-pituitary-adrenal (HPA) axis response during the calorimetric measurement, which was associated with various stress factors for the mice (i.e., social isolation, novel environment, cold stress due to lack of nesting material and enrichment items).

To ensure that potential changes in bodyweight measured during the experimental period were due to the different types of enrichment, all groups received an equal amount of oat flakes. While female C57BL/6JCrl mice (age approximately 2 months to 2 years) housed in a large semi-natural enclosure were previously shown to be heavier than those housed under CH conditions, the animals kept in cages of the same size as the CH group and provided with additional environmental enrichment items did not exhibit significant difference from the CH group ^75^. The scientific literature reports varying effects of enrichment on bodyweight, ranging from no effect to an increasing effect. For instance, it was demonstrated that environmental enrichment increased the bodyweight in female C57BL/6JCrl mice (age approximately 8–16 weeks) ^4^ or in male BALB/c mice (age approximately 8–18 weeks) ^76^. This phenomenon may be elucidated by the correlation of bodyweight with the retroperitoneal adipose tissue and bone density ^75^. Moreover, it could be argued that heightened physical exercise in enriched, more complex cages could result in a greater muscle weight. However, this hypothesis could previously not be substantiated, potentially due to the advanced age of the animals (approximately 2 years) in the study by Mieske *et al.* ^75^. In contrast, no effect of environmental enrichment on the bodyweight was found in female and male C57BL/6NTac mice (age approximately 21 weeks) ^77^, in C57BL/6JOlaHsd mice, and BALB/cOlaHsd mice (age approximately 5–8 weeks) ^78^. Additionally, cognitive tasks were shown to have a decreasing effect on the body weight in young male C57BL/6JCrl mice compared to their counterparts that were not engaged in the learning paradigms ^79^.

These discrepancies between studies may be attributed to the different types of enrichment provided and the usage of the items by the animals. Moreover, it must be noted that the amount of nesting material also plays a crucial role in bodyweight changes. Large amount of nesting material can lead to a reduction in thermoregulation activity, consequently lowering energy expenditure ^75^. A previous study showed that an increased amount of nesting material maintained a more positive energy balance and resulted in greater bodyweight in C57BL/6NCrl mice ^80^. Olsson and Dahlborn summarized in a review that mice tended to be heavier and ingested less food when provided with nesting material ^5^.

In contrast to the present study demonstrating no effect of the different types of enrichment on the resting metabolic rate, environmental enrichment was previously shown to decrease the RMR in female C57BL/6JCrl ^75^. The varying study outcomes regarding the RMR may be attributed to age differences among the mice (i.e., mice examined in the study conducted by Mieske *et al.* were 20–22 months old).

### Lockbox enrichment decreased trait anxiety-related behavior

Our study demonstrated that trait anxiety-related behavior was decreased by LE when compared to CH conditions in the Grid Exploratory Paradigm. Moreover, both LE and CH reduced trait anxiety-related behavior in comparison to SEE in the Free Exploratory Paradigm. Trait anxiety-related behavior describes an enduring personal trait stemming from past experiences of temporary fear ^81– 83^. Additionally, trait anxiety can also be influenced by genetic factors and breeding conditions ^84^. Both test paradigms, the Grid Exploratory Paradigm and the Free Exploratory Paradigm, were based on the voluntary participation of the mice, i.e., the mice voluntarily climbed on the ladder or entered the testing arena. In contrast, in the Open Field or Elevated Plus Maze Test, animals are typically compelled to participate by transferring to the maze. Since tests for trait anxiety-related behavior are generally not conducted as standard, any impact on this variable should not raise concerns among researchers. Instead, it can be viewed as a benefit for animal welfare, potentially positively influencing the affective state of the mice.

In the Grid Exploratory Paradigm, it was notable that CH decreased the total exploration time over time (i.e., when comparing values before and after the enrichment phase), contrasting with the LE and SEE, which seemed to sustain high levels of exploration. Although the latency to explore in the Grid Exploratory Paradigm was initially lower in the SEE group compared to the CH and LE groups before the enrichment phase, these differences ceased to exist after the enrichment phase. Instead, a difference between CH and LE emerged, with LE decreasing the latency to explore. Additionally, only LE decreased the latency to explore over time. The results found in the Grid Exploratory Paradigm suggested that CH increased and LE decreased trait-anxiety-related behavior. Since the only difference between the two groups was the access to the lockboxes, this type of enrichment may have affected exploration behavior. The effect on exploration may possibly be attributed to the novelty factor. A study by Bohn *et al.* gave slight hints that mice may prefer complex environments with enrichment items being regularly exchanged over, depending on the study week, complex environments with the same enrichment items and a non-enriched environment ^85^. Although LE is not comparable with environmental enrichment items providing complexity, such as houses, lying boards, or other climbing elements, lockboxes provide novel (positive) stimuli to the mice, when regularly exchanged. This may influence the expectation and consequently the behavior of the mice in tests reliant on voluntary participation, where mice have the option to either explore or refrain from exploring the testing paradigm. Since we could not analyze home cage activity and cage preference due to technical malfunctions of the MoPSS devices in our study, it remains unclear whether LE may have increased these variables, potentially supporting the results found.

In the Free Exploratory Paradigm, further interesting observations were made regarding the latency to explore. The latency to explore was reduced in the LE and CH groups compared to the SEE group. This difference may be due to the latter group being accustomed to a higher complexity and presence of hiding places in their home environment, which were absent in the testing arena. In contrast, animals from the LE and CH groups were used to less environmental enrichment items (i.e., house, tunnel, and nesting material only).

### Lockbox enrichment improved sequential problem-solving

When a 2-step lockbox was introduced to the mice following the calorimetric measurement, those from the LE group exhibited the best performance after the enrichment phase; specifically, all mice from this group successfully solved the lockbox after the 2-month enrichment phase and the number of mice able to do so increased over time in the LE group only. Additionally, mice from the LE group required the least amount of time to open it, although significance level was not achieved. This enhanced performance is likely attributable to the lockbox training, which facilitated manipulative and cognitive activities. However, the oxygen consumption rate during the lockbox trials did not differ between the study groups.

It could be hypothesized that mice from the LE group experienced less neophobia and more intrinsic interest when faced with the novel 2-step lockbox due to their familiarity with the 3D-printed items and the knowledge that a food reward was hidden inside. However, our data allowed us to reject this hypothesis because the total interaction time with the lockbox did not vary between the study groups. Furthermore, the lockbox, in both states (i.e., open and baited with an oat flake, as well as locked), was initially introduced to mice from all groups in the measurement cage to familiarize them with this new item. Therefore, it can be assumed that all mice were aware of the presence of the oat flake hidden within the lockbox.

Earlier studies have already demonstrated that C57BL/6 mice housed under enriched conditions, which include various stimulating items such as tubes, a running wheel, toys, and treats, exhibited enhanced spatial learning in the Morris Water Maze Test ^86^. Moreover, daily transfers to an enriched environment (three hours per day over two months), which provided houses, running wheels, and toys ^87^ or additionally tunnels, platforms, and pictures ^88^, have been shown to improve recognition memory in a novel-object recognition-memory task and emotional memory in a contextual fear-conditioning task in another mouse strain ^87, 88^. Given these findings, it is conceivable that LE, which encourages manipulative and cognitive activities, could similarly enhance the cognitive performance of mice. In the aforementioned studies, toys provided to the mice in enriched environments served as stimuli. If these toys were baited with treats and required specific actions to access the food, they would function on a similar principle to lockboxes.

For future studies, it would be of interest to investigate whether LE also has the potential to enhance the performance of mice in other learning and memory tasks.

### Lockbox enrichment decreased relative variability in a higher number of variables

Independent of the type of enrichment, the relative variability increased in most variables over time, i.e., values of measured parameters were higher after compared to before the enrichment phase. However, for some variables, the coefficient of variation did not increase but decreased. This trend was most often observed with LE and least often with CH, suggesting that neither lockbox nor SEE had a stronger impact on phenotypic variation. This is in line with a systematic review, demonstrating that there were no differences in the coefficient of variation across variables between environmental enrichment and control groups ^89^. Even a more complex, semi-natural environment does not increase the relative variability ^75^. In a comprehensive study, the provision of a shelter and nesting material had no consistent effect on the relative variability of various physiological parameters, leading to the conclusion that the impact was negligible ^77^. These observations were confirmed in further studies ^6, 90– 92^. It must also be considered that the mice of our study were tested at different developmental stages in life, i.e., in late adolescence and adulthood, which may have also contributed to differences in the relative variability measured before and after the enrichment phase.

### Repeatability analysis revealed impact of personality on data variances

To determine the primary contributor to variability in our data, we performed a repeatability analysis across the three days of testing for distance travelled measured in the Free Exploratory Paradigm, Open Field Test, and Elevated Plus Maze Test. In all three tests, the variances observed for locomotor activity were primarily explained by the individual animal, while group and batch did not significantly contribute to the observed variances. Our findings indicated that the individual personality of the mice played a critical role, as previously discussed as an important factor in explaining variances of data measured in mice ^60, 93, 94^. This underscored the significance of repeated testing, as explaining the variability observed in the data is a crucial step in addressing the reproducibility crisis ^60^. The emphasis on the personality of laboratory animals also opens up new avenues for enhancing animal welfare and identifying opportunities to improve the well-being of mice on an individual level ^95^.

### Habituation response in Free Exploratory Paradigm, Open Field Test, and Elevated Plus Maze Test

The Free Exploratory Paradigm, Open Field Test, and Elevated Plus Maze Test were conducted over three consecutive days to habituate the animals to the test situation, thereby circumventing an increase in variance due to novelty. A habituation response to the testing apparatus is characterized by lower levels of exploration across the days of testing. This procedure is considered to improve the reliability of the measurements ^60^.

In our study, the distance travelled in the Open Field Test decreased over the three days before but increased after the enrichment phase, suggesting a habituation response at the first time point of testing. The data generated at the second time point leave uncertainty regarding whether habituation failed or additional factors were accountable. When the mice were tested for the first and second time, they were at an age of 7–8 weeks and 17–18 weeks, respectively. It is interesting to note that other studies repeating the Open Field Test utilized mice aged no older than 10 weeks. In these studies, it was reported that locomotor activity in the Open Field Test decreased over the days of testing in male C57BL/6JCrl mice at the age of 6–7 ^60^ and female C57BL/6JRj mice at the age of approximately 10 weeks ^6^. Additionally, Bailoo *et al.* demonstrated a decrease in locomotor activity in the Open Field Test over testing days in C57BL/6 mice aged approximately 9–10 weeks ^96^. Hence, the different observations made before and after the enrichment phase may be attributed to the age of the animals. A similar observation was made in the Elevated Plus Maze Test. The distance travelled did not alter before but increased over the three days after the mice were exposed to the enrichment.

In the Free Exploratory Paradigm, the distance travelled in the testing arena increased over the three consecutive days on both time points, i.e., before and after the enrichment phase. In contrast to the Open Field Test, the mice can voluntarily choose to enter and explore the testing arena in this paradigm. Therefore, habituation is associated with a higher locomotor activity in this test situation.

The decreased coefficient of variation of distance travelled on day 3 in the Free Exploratory Paradigm and the Open Field Test suggested a successful habitation in these tests. In contrast, in the Elevated Plus Maze Test, the coefficient of variation did not decrease over the three days, which may indicate that the animals did not habituate to this testing apparatus.

### Implementation of lockbox enrichment in laboratory animal facilities

To validate the safety of the PLA material used for printing the lockboxes, a pathohistological evaluation was conducted at the end of the study, revealing no evidence of toxicopathological effects associated with PLA. As a result, exposure to lockboxes made from PLA during the temporary enrichment phase over two months (i.e., lockbox training sessions in the morning) and permanent LE phase over one month (i.e., 24 hours/day before sacrificing) did not cause any harmful effects. However, in some cases, such as studies conducted under “Good Laboratory Practice” (GLP) conditions, specific material requirements must be met. Moreover, the dimensions of the lockboxes may not be suitable for all cage sizes. In such scenarios, researcher or companies can utilize and modify our freely available lockboxes designs ^37^ to construct lockboxes in other sizes, forms, or materials.

One challenge encountered when introducing lockboxes into animal facilities is adhering to hygiene restrictions. When lockboxes are printed outside the barrier and then transported inside, they must undergo disinfection. In our study, we disinfected the lockboxes using 70% ethanol and allowed them to dry overnight. Autoclaving PLA is not feasible due to its sensitivity to heat. Instead, alternatives such as peracetic acid or hydrogen peroxide airlocks could be considered. Before implementation, compatibility tests should be conducted on small PLA samples to ensure no adverse effects occur.

An advantage of lockboxes over other enrichment strategies that facilitate cognitive and/or manipulative activities outside the home cage, such as mazes, is that they can be presented to the mice in their home cages. It is also possible to connect a playpen ^97^ including one or more lockboxes with the home cage. However, it is crucial that the mice are given “a degree of control and choice over their environment to reduce stress-induced behavior” ^3^.

### Personal subjective observation

Before the lockbox training sessions, the doors of the gate system were closed to install the lockbox for the first mouse to be trained. During this preparation time, all four mice usually attempted to access cage B and consequently queued inside or in front of the tube. The same behavior was observed when a mouse was in cage B working on the lockbox; the other mice tried to get through the door of the gate. This observation was only made as a side note and was not systematically documented. However, it may suggest that the mice were eager to gain access to cage B and subsequently the LE and/or the food reward. Unfortunately, we could not analyze home cage activity and cage preference due to technical malfunctions of the MoPSS devices in batch 2 and batch 3. Therefore, it remains unclear whether the mice may have developed a place preference for cage B as a consequence of the LE and must be investigated in future studies.

## Conclusion

Overall, we demonstrated that the lockboxes served as an effective enrichment strategy facilitating manipulative and cognitive activities. The utilization of LE positively affected the affective state and improved sequential problem-solving in female C57BL/6JCrl mice. Our findings demonstrated that implementing LE, much like other enrichment strategies, will not exacerbate the reproducibility crisis.

## Ethics and consent

Research involving animals was performed according to the guidelines of the German Animal Welfare Act and the Directive 2010/63/EU for the protection of animals used for scientific purposes. Approval for animal experimentation and maintenance of the animals was granted by the Berlin State Authority (referred to as “Landesamt für Gesundheit und Soziales”; permit number: G0249/19; date of approval: February 14, 2020). The pre-registration of the study can be found in the animal study registry (animalstudyregistry.org, doi: 10.17590/asr.0000237).

## Data Availability

Zenodo: Data and ARRIVE 2.0 checklist for the original article "Lockbox enrichment facilitates manipulative and cognitive activities for mice". https://zenodo.org/doi/10.5281/zenodo.10907380 ^98^. An xlsx file containing data for all tests presented in this article and the “ARRIVE guidelines 2.0: author checklist” are provided. Data are available under the terms of the Creative Commons Attribution 4.0 International license (CC-BY 4.0). Zenodo: Mouse Lockboxes - 3D printing files and videos (v1.0.0). https://doi.org/10.5281/zenodo.11143666 ^37^. STL files of the lockboxes as well as door systems and information on 3D printing details are given. Moreover, videos of the mice solving the lockboxes are provided. Data are available under the terms of the Creative Commons Attribution 4.0 International license (CC-BY 4.0).
