# Supplementary material for: Lockbox enrichment facilitates manipulative and cognitive activities for mice
Source: Open Res Eur. 2024 Aug 28;4:108. Originally published 2024 Jun 4. [Version 2] doi: 10.12688/openreseurope.17624.2 (PMC11384198; doi:10.12688/openreseurope.17624.2)
Supplement: Peer review history

# Reviewer report: Reviewer response for version 2

**DOI:** 10.21956/openreseurope.19899.r43585

**Published:** 2024-09-09

**Authors:** Jesse D Cushman

**Affiliations:** 1 National Institutes of Health, Durham, NC, USA

The authors have substantially improved the manuscript and I have no further concerns.  I look forward to seeing their future work on this subject.

Is the study design appropriate and does the work have academic merit?

Partly

Is the work clearly and accurately presented and does it cite the current literature?

Partly

If applicable, is the statistical analysis and its interpretation appropriate?

Yes

Are all the source data underlying the results available to ensure full reproducibility?

Partly

Are the conclusions drawn adequately supported by the results?

Partly

Are sufficient details of methods and analysis provided to allow replication by others?

Yes

Reviewer Expertise:

behavioral neuroscience, hippocampus-dependent behavior and physiology

I confirm that I have read this submission and believe that I have an appropriate level of expertise to confirm that it is of an acceptable scientific standard.

# Reviewer report: Reviewer response for version 1

**DOI:** 10.21956/openreseurope.19043.r41985

**Published:** 2024-08-05

**Authors:** Jesse D Cushman

**Affiliations:** 1 National Institutes of Health, Durham, NC, USA

In this manuscript examines the impact of an enrichment approach that involves repeated experience with lock boxes on behavioral, physiological and metabolic endpoints.  The goal was to assess whether this could be a viable option for enrichment that could improve the overall wellbeing of the mice and what impact it might have on reproducibility.  Overall, I think that the lock-boxes are an incredibly creative solution and the fact that they’ve made them publicly available so that anyone can print and construct them is a huge contribution to the field.  The videos showing the mice interacting with and solving the lock-boxes are a great resource as well.  The additional resources for gates and various other tools are very useful, too.    The study was conducted with a clear effort for rigor and reproducibility.  Unfortunately, there are several aspects of how the study was conducted, analyzed, presented, and interpreted that raise some concerns for me. I will outline those below.  I want to emphasize that I appreciate the large amount of work that was put into this and I think there is a great deal of value to the scientific community that this effort has produced.  As outlined in my final comment, I think a greater emphasis should be placed on the contribution to the field that will come from the open source tools themselves.

**Major concern:**

**The within-subjects design for tasks employed is highly problematic.** The exploration-based tasks such as open field, grid exploration and elevated plus maze are not designed to be run in a within-subjects repeated measures design.  Exploration decreases with increasing familiarity with the environment.  Reduction in exploration with increasing familiarity plays a huge role in behavior and is a direct part of many assays, such as Tonegawa’s “novel context recognition” [2] and object recognition assays.  Familiarity greatly alters the “anxiety-like” measures of open arm time and visits in the elevated plus maze.  As the environment becomes more familiar and the exploratory drive diminishes this leads to less need to explore the more threatening open arms (see Rogers and Dalvi, 1997, for example)[Ref 1].  The results in this study confirm all of this.  Figure 7a, for example, shows the expected reduction in distance travelled that would occur after averaging together the first three days of exposure to an open field vs. the second three (novel context recognition).  Figure 8C,D and E shows the expected reduction in open arm transitions that would be expected to occur in a familiar plus maze.  A better design would have been to conduct this in a between-subjects design since all three groups were run.  Little was gained from a within-subjects design and a lot was lost.   The data comparing the effects of the different housing conditions is unfortunately rather uninterpretable because of this.  The authors need to address the limitations of repeat testing due to the within-subject design limiting the interpretability of the findings.  In the discussion there is reference to the “novelty factor”, but clearly this concept needed to have been a part of the initial study design planning. 

 In addition, there are some measures where there were differences prior to the enrichment conditions, which should not be the case, and makes any interpretations highly problematic.  For example, the conclusion that the lock-box enrichment reduces trait anxiety, which I think refers to the reduced latency to explore the grid, shown in the right side of Fig 5B.  The differences in the groups before enrichment make this difficult to interpret, which is problematic because this reduction is highlighted as a major finding in the Abstract. 

 Minor:

 The descriptions of the statistical approaches are overly complex.  I think it would be better to outline the overall statistical strategy, the decision work-flow that is being employed of how or why one analysis approach was chosen over another and then provide a table or overall summary.  For most of the sections the analysis approach was the same and does not need to be repeated multiple times.  Although the statistical approach appears to have been intended to be quite rigorous, the variability in how each task was analyzed relative to the others creates an impression of inconsistency. Please provide more detail on the overall strategy and less detail on each analysis performed.  The details could be better placed in a table.  The write up of the stats also makes reading the results difficult.  It is hard to read through the statistical details in the parenthesis and still follow what is being said.  This could also be helped with a stats table.  Despite all this detail I found it hard to understand what the n’s were for each analysis, which did vary due to various technical malfunctions.  This could be included in the stats table to make it more clear.

 I found the lines in the figures indicating statistical significance a bit difficult to follow.  Perhaps details could be put in the figure legend?

 It is unfortunate that males could not be included. What is the plan to integrate the analysis of the males in the future.  Will sex differences be examine directly in any way?

 Figure 6 doesn’t really help show anything useful (other than the clear reductions in exploration that occur with increasing familiarity).  Data needs to be separated by group to be useful. 

 Stating that training with the lockboxes improved sequential problem solving might be misleading, as the training involved the same task.  Wording it this way makes it sound like a different cognitive assay was run and the lock box enrichment improved performance on a different task.  The results indicate that training and experience with lock boxes improved performance with solving lock boxes.  This should be made more clear.

 General question: How would lock boxes be implemented at a vivarium level?  They would need to be re-baited and each mouse would need the highly labor intensive individualized exposure that was used here.  Would experimenters or animal techs re-bait the lock boxes?  Could the lock boxes be designed to be repeatable somehow?  It’s not clear to me how these lock boxes (as amazing as they are) could really be implemented at scale.

 The final conclusion of the paper is quite conservative and, I think, defensible given the data: “ *Our findings demonstrated that* *implementing LE, much like other enrichment strategies, will* *not exacerbate the reproducibility crisis.”* To my mind, however, what I find most exciting about the paper is the open source tools it makes available to the community. I would have liked to see more emphasis on solving the lock boxes as a task in its own right.  I could see this as being very useful in cognitive assessments in many modelling situations.  Perhaps the emphasis of the paper could highlight this more?  Otherwise, I think the greatest strength of the paper – the introduction of an innovative open source tool, is rather lost in large amount of data that is difficult to interpret.

Is the study design appropriate and does the work have academic merit?

Partly

Is the work clearly and accurately presented and does it cite the current literature?

Partly

If applicable, is the statistical analysis and its interpretation appropriate?

Yes

Are all the source data underlying the results available to ensure full reproducibility?

Partly

Are the conclusions drawn adequately supported by the results?

Partly

Are sufficient details of methods and analysis provided to allow replication by others?

Yes

Reviewer Expertise:

behavioral neuroscience, hippocampus-dependent behavior and physiology

I confirm that I have read this submission and believe that I have an appropriate level of expertise to confirm that it is of an acceptable scientific standard, however I have significant reservations, as outlined above.

## References

[1] : Anxiety, defence and the elevated plus-maze. *Neurosci Biobehav Rev* .1997;21(6) : 10.1016/s0149-7634(96)00058-9 801-10 10.1016/s0149-7634(96)00058-9 9415905

[2] : Locus coeruleus input to hippocampal CA3 drives single-trial learning of a novel context. *Proc Natl Acad Sci U S A* .2018;115(2) : 10.1073/pnas.1714082115 E310-E316 10.1073/pnas.1714082115 29279390 PMC5777050

# Response

**Published:** 2024-08-15

**Authors:** Katharina Hohlbaum

**Affiliations:** German Federal Institute for Risk Assessment (BfR), German Centre for the Protection of Laboratory Animals, Germany

We thank the reviewer for taking the time to read and comment on our work, which greatly enhanced the value of our manuscript. Below we answered point-by-point to all major and minor concerns:

**Major concerns:**​​​​​​​

 1. **Within-subjects design: **There is a significant reproducibility crisis in research, and it is imperative for all researchers to consider ways to enhance data reliability. One strategy is to identify methods to reduce data variability. A high variability in the data can obscure group/treatment effects, potentially leading to a misinterpretation of the results. If animals are habituated to the test situation and the variability decreases, the data will be more reliable, which was previously proposed by Rudeck at al. (2020). Therefore, the Free Exploratory Paradigm, Open Field Test, and Elevated Plus Maze Test were conducted over three consecutive days. Table 2 shows that the coefficient of variation decreased in the Free Exploratory Paradigm and Open Field Test over the three days, demonstrating that repeated testing in these two paradigms was an efficient strategy to reduce data variability. However, we recognize the reviewer’s criticism about repeated testing, which should be taken into account. We added an additional paragraph to the discussion section on “Habituation response in Free Exploratory Paradigm, Open Field Test, and Elevated Plus Maze Test” and pointed out that the repeated testing in the Free Exploratory Paradigm, Open Field Test, and Elevated Plus Maze Test can limit the interpretability of the results. Rudeck J, Vogl S, Banneke S, *et al.*: Repeatability analysis improves the reliability of behavioral data. *PLoS One.* 2020; **15**(4): e0230900.​​​​​​​

 2. **Baseline values:** We hope that the reviewer acknowledged that we examined and reported “baseline” values (i.e., before enrichment phase). Unfortunately, this is still not standard in all animal behavior studies. We would like to address a possible misunderstanding regarding the statement that “there are some measures where there were differences prior to the enrichment conditions”. There is only one variable with significant baseline differences between the groups, which was transparently pointed out in the results section on “Latency to explore” and in the discussion section on “Lockbox enrichment decreased trait anxiety-related behavior". In brief, mice assigned to CH and LE showed a higher latency to explore compared to mice assigned to SEE before the enrichment phase. After the enrichment phase, these differences ceased to exist and only a difference between CH and LE was detected (i.e., the two groups that did not differ at baseline). When comparing the latency to explore before and after the enrichment phase within the groups, the values significantly decreased in the LE group only. In response to the reviewer's concerns, we will focus our analysis on the within-group comparison of the Grid Exploratory Paradigm to draw the conclusion that lockbox enrichment reduces trait anxiety-related behavior. Additionally, this conclusion is further supported by the results from the Free Exploratory Paradigm. The abstract and discussion section on “Lockbox enrichment decreased trait anxiety-related behavior” were revised accordingly.

**Minor concerns:**

**1. Statistical analysis: **As suggested by the reviewer, more general information on the “overall statistical strategy” was added to the paragraph on “Statistical analysis” and an additional table providing an overview of the statistical strategy (Table 1) was created. Table 1 provides details on the statistical analysis of each variable. If other factors than described in the section on statistical analysis influenced the choice of model, the rationale is given in Table 1. In the scope of transparency and reproducibility, the authors would prefer not to remove any further details on the statistical analysis form the manuscript. For researchers not familiar with LMM/GLMM it may not be clear that it is important to compare model fits and chose the most suitable model for the dataset. Depending on the fixed/random factors, distribution of data/residuals, and model fit comparisons, it may occur that not the same model can be used for analyzing the different datasets of the study. This may be the reason why the reviewer had the impression that “the variability in how each task was analyzed relative to the others creates an impression of inconsistency”. If the reviewer is more specific about this criticism and suggests how to improve the statistical analysis, the authors will be happy to discuss his suggestions.

**2. Symbols indicating significant differences in figures: **We are sorry that the meaning of lines and asterisks were not clear. To provide further clarification, we added an explanation to the legends of figure 5 and 8: An asterisk (*) indicates a significant difference between two groups before or after the enrichment phase, or a significant difference within a group before and after the enrichment phase (details are given in the results section).

**3.Male animals: **As explained in the manuscript, the study planning included both sexes but so far were have only been able to examine females due to the time-consuming nature of the study. We emphasized this limitation in the article: “The study planning included both sexes. Due to the time-consuming nature of the study, thus far, we have only been able to examine female animals, which must be considered as limitation of the present article.”

**4.Figure 6: **As suggested by the other reviewer, Figure 6 was moved to the supplementary material: Hohlbaum, K., Andresen, N., Mieske, P., et al.: Extended data of the article "Lockbox enrichment facilitates manipulative and cognitive activities for mice": Supplementary Figure [Extended data]. Zenodo. 2024. https://doi.org/10.5281/zenodo.13269372https://doi.org/10.5281/zenodo.13269372 Data were not separated by groups in this figure since differences in the distance traveled between the days were of interest. To compare the data from the present study with those generated by Rudeck et al. (2020), the same statistical analysis was applied.

**5. Wording “sequential problem solving with lockboxes”:** We apologize for the misleading wording in the abstract and conclusion. After reading the discussion section of the manuscript, it should be clear that the authors were referring to sequential problem-solving with lockboxes. To clarify this, the corresponding sentences in the abstract and conclusion were rephrased.

 However, the authors also would like to point out that the training of the LE group did not involve the same lockbox as used during the calorimetric measurement when a lockbox was presented to mice of all groups (i.e., CH, SEE, and LE). Mice of the LE group were trained on the lockboxes shown in Figure 3a and 3b. At the end of the calorimetric measurement, a novel lockbox was presented to the mice (Figure 3c).

**6. General question on implementation of lockboxes a vivarium level: **First, it is essential to recognize our moral responsibility and duty to continuously refine the experimental and housing conditions of laboratory animals. This obligation applies to everyone working with laboratory animals. The Directive 2010/63/EU stipulates that the enrichment strategies in establishments shall be regularly reviewed and updated. We can consider various strategies for implementing lockboxes in everyday work of an animal facility. However, since the strategies depend on several factors (such as space, housing conditions, hygiene levels, etc.), each establishment must develop a feasible approach tailored to their specific circumstances. As described in the discussion section, lockboxes can either be added to the home cages or playpens which are connected to the home cage. If short individual lockbox sessions in the home cage, similar to the temporary lockbox enrichment introduced in this manuscript, are not feasible, an alternative could be to provide several single mechanisms in the group cage, which can be rebaited or changed during the daily inspection, similar to the permanent lockbox enrichment described here. Our lab is also working on an automated lockbox. If the box automatically locks the mechanisms and is rebaited after being solved, it would benefit both the animals and the caretakers. We added these thoughts to the discussion section on “Implementation of lockbox enrichment in laboratory animal facilities”.

**7.Conclusion of the paper:** The present manuscript is part of an extensive study divided into several substudies. In the substudy presented in this article, we focused on phenotyping and animal welfare; another substudy analyzes the problem-solving strategies of mice in lockbox sessions using computer vision methods, which may be of particular interest to researchers in the field of behavioral neuroscience. The preprint of this substudy can be found here (Boon et al. 2024): https://doi.org/10.1101/2024.07.29.605658. We agree that lockbox tasks can be useful in cognitive assessment, as highlighted in a review by Lang et al. (2023). However, when introducing lockboxes as a novel type of enrichment or task for cognitive assessment, understanding their impact on the phenotype of mice is crucial. Discussions on the implementation of enrichment strategies often include concerns that data variability will increase and/or that the animals' phenotype will be significantly affected. Consequently, animals that would naturally live in complex habitats are still housed in small, barren cages, which negatively impacts their well-being. Therefore, the data presented in this manuscript are of utmost importance to this discussion and need to be shared with the scientific community in their current detail. We hope this will contribute to improve the welfare of laboratory mice in the future. According to your advice, we highlighted the points mentioned above (i.e., open-source tool, novel approach in behavioral neuroscience) in the abstract, discussion, and conclusion. Marcus N. Boon, et al. Mechanical problem solving in mice. bioRxiv 2024.07.29.605658; doi: https://doi.org/10.1101/2024.07.29.605658. Lang B, Kahnau P, Hohlbaum K, *et al.*: Challenges and advanced concepts for the assessment of learning and memory function in mice. *Front Behav Neurosci.* 2023; **17**: 1230082.

# Reviewer report: Reviewer response for version 1

**DOI:** 10.21956/openreseurope.19043.r41692

**Published:** 2024-07-12

**Authors:** Rasneer Sonia Bains

**Affiliations:** 1 Mary Lyon Centre at MRC Harwell, Oxfordshire OX11 0RD, UK

The manuscript is well thought out, and the experimental design, analysis and reporting has been done in line with best practices and reproducibility in mind. I congratulate the authors on this exemplary effort. If I may, I would like to recommend a few minor changes that may help the readers along as it is a very comprehensive manuscript.

1. Of the two main conclusions: adding enrichment won't alter the phenotype and that the lock box enrichment facilitates manipulative and cognitive activities, only the latter is clear from the outset. It would be good to state both of these clearly at the beginning.
2. To improve the legibility of the Box and Whisker plots, may I suggest using a single legend explain the colours, it will really help the readers.
3. Figure 6 could reasonably be moved to supplementary data.
4. When describing the results the authors refer to comparisons that are not shown in the figure, just before mentioning the figure, this is a bit confusing, e.g. " **Total interaction time with the lockbox:** While the group did not affect the interaction time with the lockboxes (F _2, 6_ = 0.17, p = 0.844), time had a significant effect (F _1, 35_ = 22.58, p < 0.001) in the LMM ( Figure 9c). The mice interacted for a longer time duration with the lockbox after compared to before the enrichment phase." Please either show these data or move "(Figure9c)" to the place in the text where it relates to the data shown. This is also true of most text references to Figure 8.
5. Legend for Figure 5, please replace "und" with "and" :) 
6. When describing the results from DLC the authors mention that data from some animals may not be included, would it be possible to say how many animals per dataset were not included?
7. Thank you for sharing the interrater reliability data. It is refreshing to see such attention to best practices.
8. The two statements below are a bit confusion, please could you consider rephrasing them:
  1. **Total interaction time with the lockbox:** While the group did not affect the interaction time with the lockboxes (F _2, 6_ = 0.17, p = 0.844), time had a significant effect (F _1, 35_ = 22.58, p < 0.001) in the LMM ( Figure 9c). The mice interacted for a longer time duration with the lockbox after compared to before the enrichment phase.
  2. **Interaction time with the lockbox until solved:** Among the mice that were able to solve the lockbox, time significantly affected the interaction time with the lockbox until it was solved (F _1, 19.35_ = 12.67, p = 0.002) in the LMM ( Figure 9d). Group had no significant effect (F _2, 26.38_ = 0.53, p = 0.594). The interaction time with the lockbox until solved was decreased after compared to before the enrichment phase.
9. It is not entirely clear from statement "The FCM values were higher after the exposure to the different types of enrichment compared to baseline." on Page 26 if this is back up with stats, please could you rephrase.

While the authors explain why the data only relates to one sex, it would have been good to see the male data. I take it on good faith that the authors will be publishing these data soon. I very much look forward to reading that manuscript too.

Is the study design appropriate and does the work have academic merit?

Yes

Is the work clearly and accurately presented and does it cite the current literature?

Yes

If applicable, is the statistical analysis and its interpretation appropriate?

Yes

Are all the source data underlying the results available to ensure full reproducibility?

Yes

Are the conclusions drawn adequately supported by the results?

Yes

Are sufficient details of methods and analysis provided to allow replication by others?

Yes

Reviewer Expertise:

Behavioural Phenotyping. Experimental design and Analysis.

I confirm that I have read this submission and believe that I have an appropriate level of expertise to confirm that it is of an acceptable scientific standard.

# Response

**Published:** 2024-08-15

**Authors:** Katharina Hohlbaum

**Affiliations:** German Federal Institute for Risk Assessment (BfR), German Centre for the Protection of Laboratory Animals, Germany

We thank the reviewer for taking the time to read and evaluate our article and for appreciating our work. The comments provided were highly constructive and have helped to improve the manuscript significantly. Below we answered point-by-point to all minor concerns.

1. **Stating main conclusions clearly at the beginning: **You are absolutely right. We revised the abstract, conclusion, and first paragraph of the discussion section to emphasize this aspect.
2. **Color legends in figures:** The figures were revised accordingly.
3. **Figure 6:** Figure 6 was moved to the supplementary data: Hohlbaum, K., Andresen, N., Mieske, P., et al.: Extended data of the article "Lockbox enrichment facilitates manipulative and cognitive activities for mice": Supplementary Figure [Extended data]. Zenodo. 2024. https://doi.org/10.5281/zenodo.13269372
4. **Order of figure citations: **This does seem to be confusing. As suggested, the "figure names" were moved to locations in the text where they more closely correspond to the data being presented.
5. **Typo: **Thanks for spotting this typo :)
6. **Numbers of animals:** The number of animals per dataset is provided in the figure and table legends. To make this information more accessible to the reader, we have created an additional table (Table 1) that includes the number of animals per dataset, along with an overview of the statistical analyses. The section on the statistical analysis had to be shortened due to concerns of the other reviewer. He stated that the “descriptions of the statistical approaches are overly complex” and suggested creating this table.
7. **Interrater reliability:** Thanks a lot for appreciating!
8. **Rephrasing:**
  1. In the LMM, group had no significant effect (F2, 6 = 0.17, p = 0.844) (Figure 9c) but time had a significant effect on the interaction time with the lockboxes (F1, 35 = 22.58, p < 0.001). The mice spent more time interacting with the lockboxes after the enrichment phase compared to before it.
  2. Among the mice that were able to solve the lockbox, group did not significantly affect the interaction time with the lockbox until it was solved in the LMM (F _2, 26.38_ = 0.53, p = 0.594) ( Figure 9d). Time had a significant effect (F _1, 19.35_ = 12.67, p = 0.002). The interaction time with the lockbox until it was solved decreased after the enrichment phase compared to before it.
9. **Rephrasing: **The sentence referred to the finding that the fixed factor “time” significantly affected the values (F _1, 35_ =32.89, p < 0.001). The sentence was rephrased to clarify its meaning: “Independent of the group, the FCM values were higher after the exposure to the different types of enrichment compared to baseline.”
